# Library-Derived Peptide Aggregation Modulators of Parkinson’s Disease Early-Onset α-Synuclein Variants

**DOI:** 10.1021/acschemneuro.2c00190

**Published:** 2022-05-25

**Authors:** Kathryn J. C. Watt, Richard M. Meade, Robert J. Williams, Jody M. Mason

**Affiliations:** Depart of Biology and Biochemistry, University of Bath, Claverton Down BA2 7AY, United Kingdom

**Keywords:** peptides, amyloid aggregation, early-onset Parkinson’s disease

## Abstract

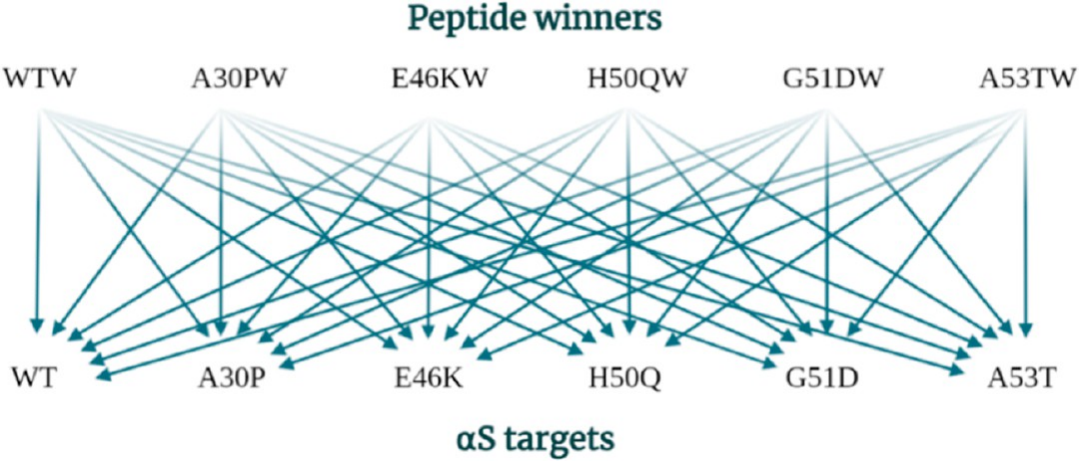

Parkinson’s Disease (PD) is characterized by the accumulation of Lewy bodies in dopaminergic neurons. The main protein component of Lewy bodies, α-synuclein (αS), is also firmly linked to PD through the identification of a number of single point mutations that are autosomal dominant for early-onset disease. Consequently, the misfolding and subsequent aggregation of αS is thought to be a key stage in the development and progression of PD. Therefore, modulating the aggregation pathway of αS is an attractive therapeutic target. Owing to the fact that all but one of the familial mutations is located in the preNAC 45–54 region of αS, we previously designed a semi-rational library using this sequence as a design scaffold. The 45–54 peptide library was screened using a protein-fragment complementation assay approach, leading to the identification of the 4554W peptide. The peptide was subsequently found to be effective in inhibiting primary nucleation of αS, the earliest stage of the aggregation pathway. Here, we build upon this previous work by screening the same 45–54 library against five of the known αS single-point mutants that are associated with early-onset PD (A30P, E46K, H50Q, G51D, and A53T). These point mutations lead to a rapid acceleration of PD pathology by altering either the rate or type of aggregates formed. All ultimately lead to earlier disease onset and were therefore used to enforce increased assay stringency during the library screening process. The ultimate aim was to identify a peptide that is effective against not only the familial αS variant from which it has been selected but that is also effective against WT αS. Screening resulted in five peptides that shared common residues at some positions, while deviating at others. All reduced aggregation of the respective target, with several also identified to be effective at reducing aggregation when incubated with other variants. In addition, our results demonstrate that a previously optimized peptide, 4554W(N6A), is highly effective against not only WT αS but also several of the single-point mutant forms and hence is a suitable baseline for further work toward a PD therapeutic.

## Introduction

The misfolding and subsequent aggregation of the protein α-synuclein (αS) is associated with a range of diseases known as synucleinopathies, the most common of which is Parkinson’s Disease (PD), but also includes Dementia with Lewy Bodies and multiple system atrophy. PD is the second most common neurodegenerative disease, currently affecting ∼200 000 people in the UK; however, owing to an aging population, this number is expected to rise in the coming years.^1^ PD is predominantly a movement disorder, with the most prevalent symptoms including rigidity, tremor, and slowness of movement; however, there is also a wide range of non-motor symptoms associated with PD, and further, up to ∼75% develop dementia.^2,3^ The current treatments for PD provide symptomatic benefit through targeting the dopamine deficit (e.g., levodopa); however, there is an unmet need to develop disease-modifying therapeutics that can slow disease progression.

αS was first linked to PD through the identification of a missense mutation in the gene encoding αS (*SNCA*), resulting in the single point-mutation A53T.^4^ In the same year, αS was also determined to be the main protein component of Lewy bodies, providing an unequivocal link between αS and PD.^5^ Subsequently, further missense mutations have been identified, along with gene duplications and triplications, all of which often result in early-onset PD.^4,6−16^ αS is a 140-amino acid, intrinsically disordered protein, and is predominantly located at synapses where it is thought to be involved in a range of functions including synaptic plasticity and regulating synaptic vesicle release at nerve terminals via lipidic interactions, although its precise role is not yet fully understood.^17−19^ While natively unfolded, αS forms an α-helical structure in the presence of membranes.^20−22^ However, in PD, αS misfolds to form a range of oligomers before forming extended β-sheet amyloid fibrils.^23−25^ This aggregation pathway is extremely complex and not yet fully understood: a range of different on- or off-pathway oligomers and polymorphs of αS are able to be formed, with an off-pathway “toxic oligomer” currently thought to be a likely contributor to the onset of PD.^25−28^ Moreover, association of αS with free phospholipids in the aqueous phase can result in the formation of an αS-lipid complex (the lipid-chaperone hypothesis).^29^ This αS-lipid complex is able to disrupt membranes, leading to cell toxicity. Ultimately, these aggregates accumulate in the *substania nigra pars compacta* to form Lewy bodies—the pathological hallmark of PD.^5^

Modulation of the αS aggregation pathway is an attractive option toward developing a disease-modifying therapeutic for PD. However, to date, the development of small-molecule inhibitors of αS aggregation has proved futile due to the wide range of complex protein–protein interactions (PPIs) that are formed during the aggregation pathway. These interactions are broad and shallow and therefore not typically suitable for the design of small-molecules that are more suitable for distinct binding pockets. Therefore, peptides are uniquely placed as a logical alternative to fit these otherwise “undruggable” targets.^30,31^ Moreover, peptides exhibit a number of advantages over small molecules and larger antibodies including avoidance of immunogenicity (when short) since they fall below the threshold required for presentation and are target-specific due to the high number of interactions with the target. As natural products, they are less likely to be toxic, and can be quickly and cheaply synthesized to high purity. There are some limitations with peptides that traditionally include susceptibility to protease degradation, high clearance rates, low oral bioavailability, low membrane permeability, and high flexibility that can result in low binding affinity. However, these issues are continually being addressed and potential solutions being provided, such as peptidomimetics (N-methylation, peptoids, non-natural amino acids, and retro-inverso peptides), constraints (cyclization and stapled peptides), and cell-penetrating peptides or lipidic appendages.^32,33^

**Figure 1:**
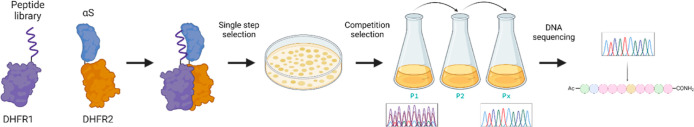
PCA overview. The peptide library is fused to one-half of mDHFR (murine dihydrofolate reductase), and the αS (1–140) target is bound to the other half. If a library member binds to the αS target, the DHFR recombines, leading to cell survival under selective conditions. PCA winners with the highest efficacy are identified via competition selection through successive passages in liquid media. DNA sequencing of the library pools for each passage leads to identification of the dominant peptide sequence, and hence identification of the PCA winner peptide. Ultimately, a single sequencing result is obtained for each target.

**Figure 2:**
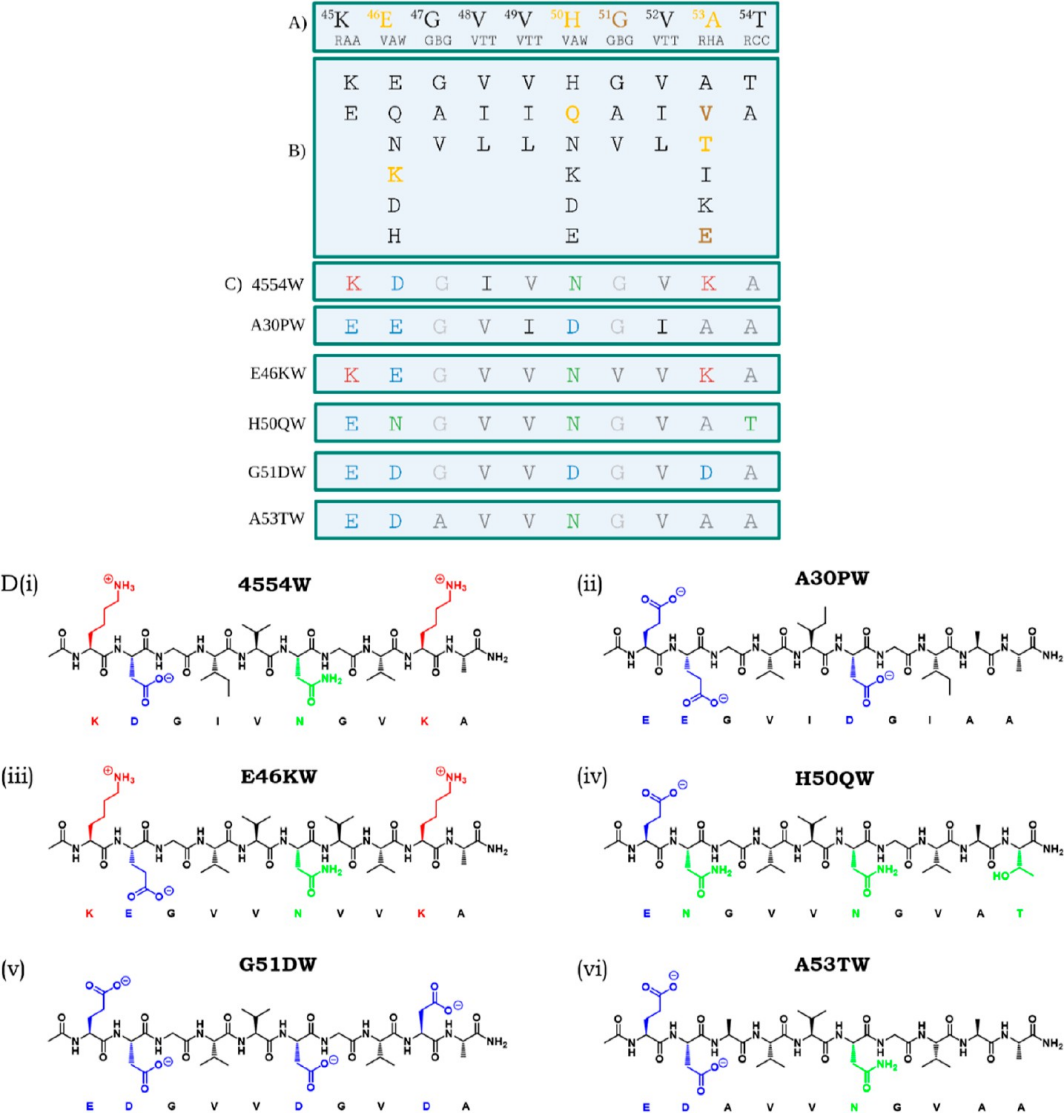
Library screen, sequences, and structures of the respective PCA winner peptides. (A) WT αS residues 45–54 that constitute the library template region. The library contained both wt options as well as conserved changes and familial mutations known at the time of original library design (shown in yellow). Subsequently identified mutations are shown in brown. Degenerate codons of the library construction are shown below the sequence (R = A/G, V = A/C/G, W = A/T, B = C/G/T, and H = A/C/T). (B) Amino acid options for each position, generating a library of 209 952 members. (C) Original PCA winner peptide (4554W) sequence and respective mutant winner peptides A30PW, E46KW, H50QW, G51DW, and A53TW. (D) Structures of the respective peptide winners in black representing hydrophobic residues, red are positively charged, blue are negatively charged, and green are polar.

Previous work in our group identified the peptide 4554W via an intracellular protein-fragment complementation assay (PCA) (Figure 1).^34^ PCA works by recombining a split enzyme, such that a peptide is identified if it binds to the target (αS). Intracellular selection means that in addition to αS binding, there must also be a favorable change in cytotoxicity of the target in order for a given library member to become enriched during consequent competition selection (Figure 1). Furthermore, PCA selects peptides that are protease-resistant, soluble, and non-toxic. In this case, 4554W was shown to be successful in reducing the extent of αS aggregation and recovering cytotoxicity. Specifically, 4554W inhibits αS aggregation by inhibiting the lipid-induced primary nucleation step.^35^ The 209 952-member library (Figure 2) was based on the preNAC region 45–54 of WT αS due to most of the mutations associated with familial PD being located within this region (E46K, H50Q, and A53T at the time of original design, with G51D, A53E, and A53V being subsequently identified). Much of the previous work has focused on the 71–82 region.^36^ The presence of these mutations cements the importance of the preNAC region for αS toxicity in PD, through their unambiguous importance to the formation and modulation of the PPI formations (both inter- and intramolecular) formed during αS aggregation in PD pathology.

Here, we build on previous work by expanding the library screen to include five of the early-onset variants (A30P, E46K, H50Q, G51D, and A53T) as the target protein. Following identification of the 5 PCA peptide hits (A30PW, E46KW, H50QW, G51DW, and A53TW), the peptide winners were characterized both against their intended target and also against the other αS variants through experiments including the ThT-monitored aggregation assay, circular dichroism (CD) (to monitor changes to global secondary structure), transmission electron microscopy (TEM) (to monitor changes to fibril load and morphology), photo-induced cross-linking of unmodified protein (PICUP) cross-linking (to monitor changes to the distribution of oligomer formation), and MTT (to assess protection against αS-induced toxicity). Expressing and targeting αS variants associated with early-onset disease was undertaken to generate a more stringent screening assay toward an improved peptide that can serve as a baseline for further development for therapeutics effective against WT (affecting the majority of late-onset PD), but clearly also one or more of the more toxic forms associated with early-onset PD.

## Results and Discussion

### Identification of Peptides

We previously published the identification of the peptide 4554W that is effective in inhibiting the earliest stages of αS aggregation.^34,35^ This peptide was identified using a PCA library screen (Figure 2), which used αS 45–54 as the design scaffold. This region was selected because it contained all but one (A30P) of the single point mutations associated with familial PD (E46K, H50Q, and A53T).^4,7,15,16,37^ Subsequent to this initial work, further point mutations associated with familial PD have been identified including G51D, A53E, and A53V.^8,10,11,38,39^ A53E and A53V were incorporated into the original library design, but G51D and hence D/E residues have not been included. 4554W was found to be effective in a dose-dependent manner using cell toxicity assays (MTT), ThT aggregation assays, CD, and microscopy (atomic force microscopy).^34^

However, although based on the familial mutations of αS, the library was not originally screened against these αS variants. Here, we screened this same library against five of the αS variants associated with early-onset PD (A30P, E46K, H50Q, G51D, and A53T), resulting in the identification of five unique peptide sequences (Figure 2). The family of peptide winners was synthesized by solid-phase peptide synthesis (SPPS) and characterized using a range of experiments including ThT aggregation assays, CD, PICUP, TEM, and MTT cytotoxicity assays. Discussed in the following sections are the characterization against the respective targets and their effectiveness against the other αS variants.

### Peptides Reduce Aggregation of Their Respective Target via Lipid-Induced Primary Nucleation

**Figure 3:**
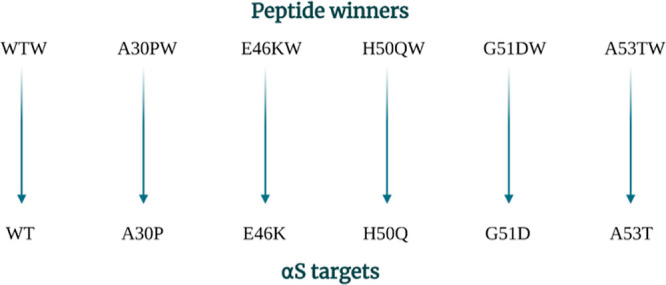
Characterization of peptide winners against their respective targets. Effectiveness of each peptide winner (WTW, A30PW, E46KW, H50QW, G51DW, and A53TW) in modulating the aggregation of the respective αS target (WT, A30P, E46K, H50Q, G51D, and A53T) is investigated.

In order to determine the ability of each PCA-derived peptide to reduce the aggregation of its respective target (Figure 3), we employed lipid-induced primary nucleation assays.^21^ Primary nucleation is the earliest stage of the aggregation pathway, and thus inhibition of this step is arguably the most important and most promising for developing a therapeutic that is effective in the very earliest stages of the disease. We previously determined that 4554W inhibits this pathway,^35^ and therefore, we focused our efforts to characterize the efficacy of the αS variant-PCA peptides (Figure 2) against their respective targets in the same way. For this assay, we used DMPS as our model lipid since αS preferentially binds to negatively charged lipids (through interaction with the positively charged lysine residues of αS); phosphatidylserine (PS) has been shown to be an abundant component of synaptic vesicle membranes; the PS concentration levels increase by more than a third in individuals with incidental PD; and DMPS is associated with αS-facilitated synaptic vesicle docking.^20,40−44^ Furthermore, we used an [αS/DMPS] ratio of 1:2 for our primary nucleation ThT assays since this has been previously found to result in increased rates of αS aggregation. In contrast, at significantly higher ratios, αS aggregation becomes inhibited (i.e., at a ratio where αS predominantly exists in the lipid-bound α-helical state).^21^ The concentration of αS in healthy neurons is predicted to be in the region of 70–140 μM; therefore, here we have utilized a concentration of 100 μM αS to align with physiologically relevant concentrations.^45^

**Figure 4:**
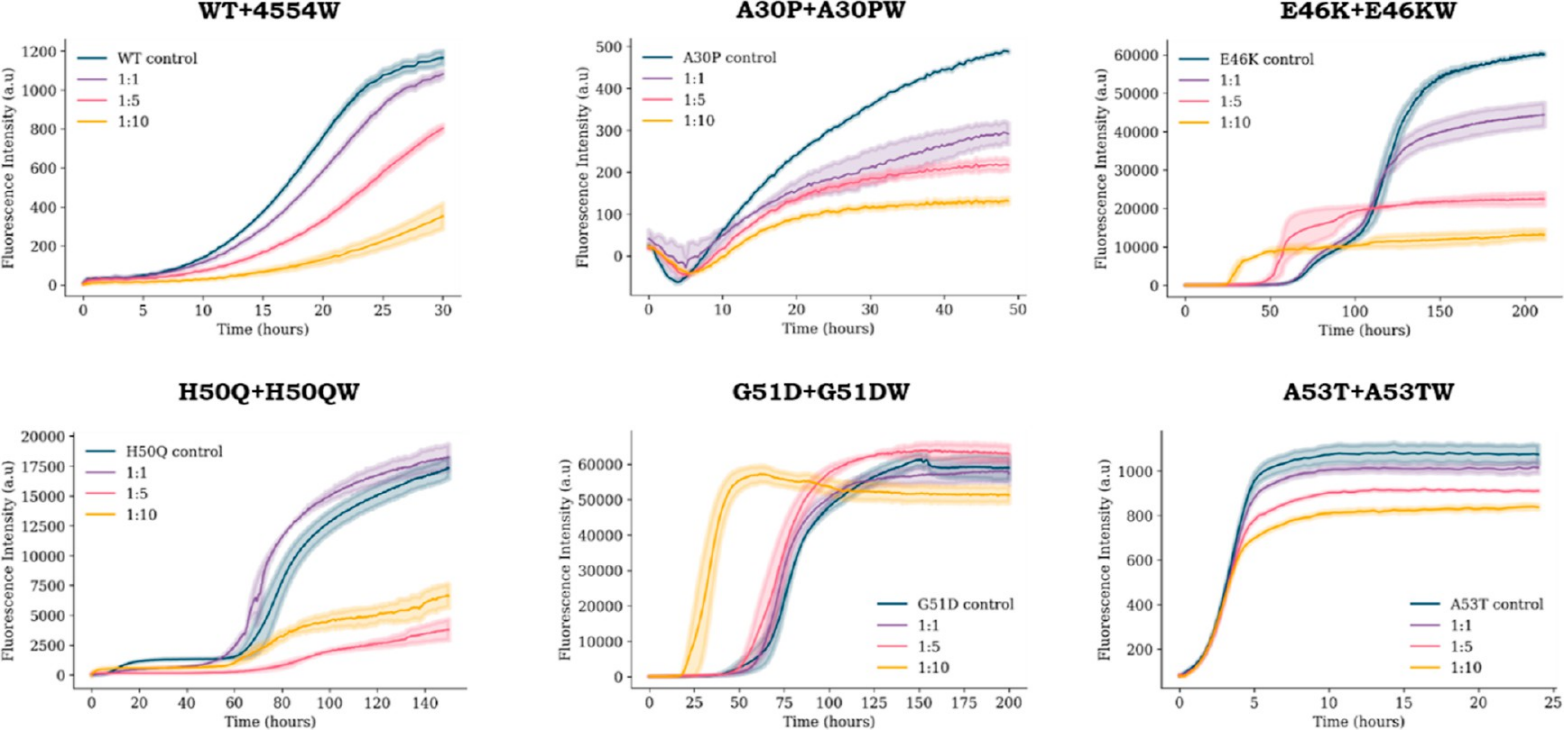
ThT dose–response for each αS variant and the respective PCA winner peptide. Each αS variant [100 μM (dark blue)] was incubated with the respective peptide winner [100 μM (purple), 500 μM (pink), or 1000 μM (yellow)], DMPS SUVs (200 μM) and ThT (50 μM) in 20 mM sodium phosphate buffer, pH 6.5 at 30 °C under quiescent conditions until the ThT signal plateaued (up to 250 h). The average of three repeats is shown with the standard error.

As summarized in Figure 4, a dose–response for each peptide winner against their respective target was observed. However, the apparent effectiveness of the peptides varied widely, from A30PW (at a 10:1 M excess) resulting in a reduction in A30P aggregation of 72% down to A53TW only reducing the aggregation of A53T by 23%. On the other hand, G51DW and E46KW markedly reduced the lag time for aggregation of their respective αS variants, with G51DW resulting in a 61% reduction in lag time (i.e., quicker to aggregate) and a negligible (13%) reduction in the overall ThT intensity, whereas E46KW resulted in a similar 72% reduction in lag time and also had a remarkable effect in reducing the extent of aggregation by reducing the ThT signal by 78%.

These differences indicate that the mechanisms under which αS variants aggregate may differ (e.g., E46K undergoes a multi-step aggregation pathway—Figure 4), and the way in which the peptides modulate this aggregation pathway also vary. For example, in order for an increase in ThT to be observed for E46K, G51D, and H50Q, substantially longer incubation times are required than for WT, A30P, and A53T (e.g., up to 250 h vs 48 h). This may indicate that this is no longer purely monitoring primary nucleation, but it may progress to other lipid-induced aggregation mechanisms. However, it is clear that these peptides modulate the aggregation pathway for all αS variants.

### αS Variants Form Two Distinct Groups of Global Secondary Structure under Lipid-Induced Aggregation Conditions

**Figure 5:**
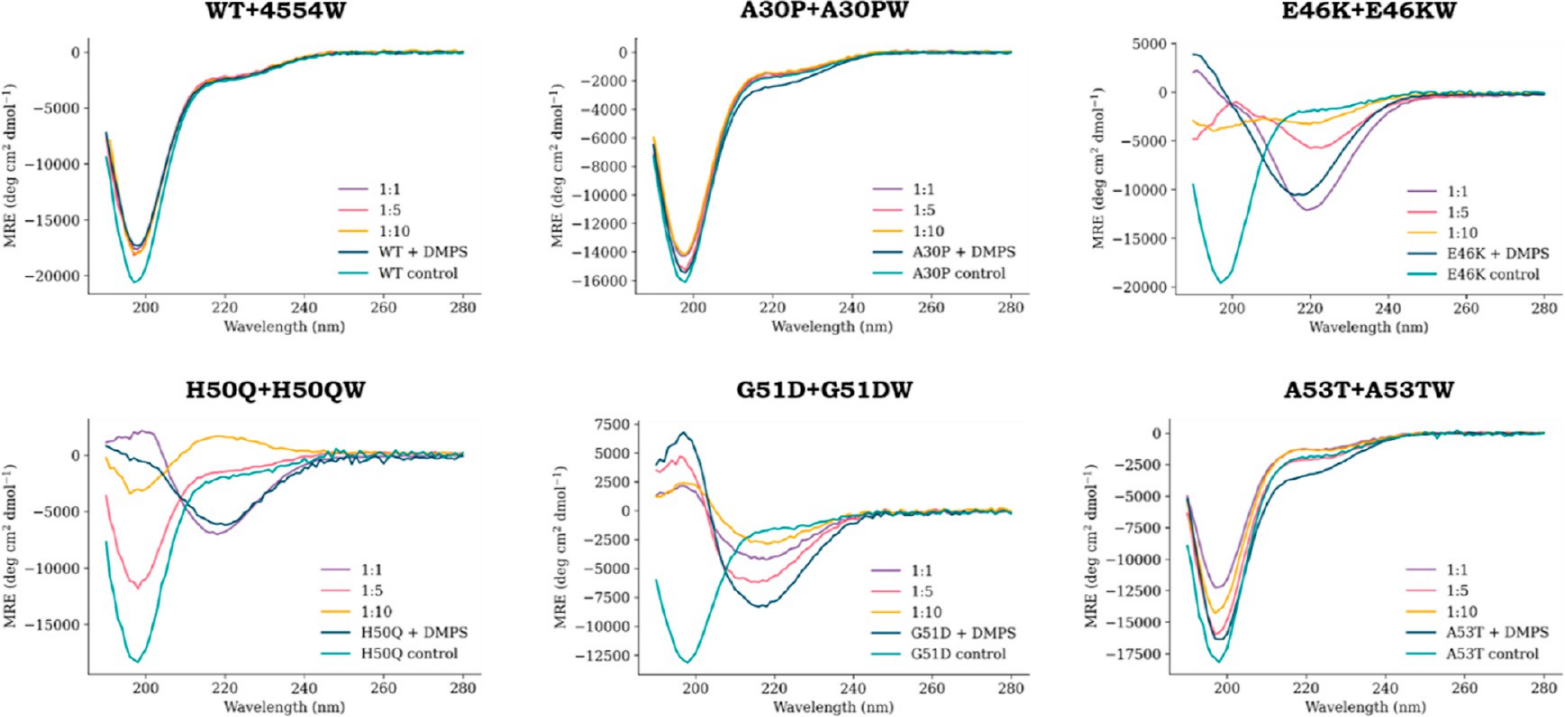
CD dose–response for each αS variant and the respective PCA winner peptide. ThT end-point samples for each αS variant (WT, A30P, E46K, H50Q, G51D, or A53T) incubated with the respective PCA winner peptide (4554W, A30PW, E46KW, H50QW, G51DW, and A53TW) were diluted 10-fold to achieve an αS concentration of 10 μM. The spectra show that the average of three repeats is blanked against the assay buffer (20 mM sodium phosphate, pH 6.5), and the respective peptide control has been subtracted in order to view the changes to the αS structure.

To determine if the dose–response observed in the ThT aggregation assays (Figure 4) corresponds to a respective change in the global protein secondary structure, we analyzed the end-point samples with CD. In particular, we were interested to investigate if any of the peptides resulted in a reduction in β-sheet, or conservation of the monomeric random-coil structure, of the αS variants. In Figure 5, the CD spectra for each of the αS mutants and the dose–response for the respective winner peptide from the end point of the ThT aggregation assays are shown. Notably, WT, A30P, and A53T do not show a significant change in the secondary structure throughout the aggregation pathway, with the global secondary structure remaining predominantly random coil. However, for A30P and A53T, there is a slight increase in β-sheet character with the αS control sample, which was reduced in the presence of the peptides. A much greater extent of β-sheet formation was observed for E46K, H50Q, and G51D, with each of the respective peptides resulting in a reduction in the extent of β-sheet formation in a dose-dependent manner. This was most stark for H50Q, where a significant amount of random-coil structure was conserved with H50QW.

The large extent of β-sheet character observed for E46K, H50Q, and G51D corresponds to a ThT intensity that is an order of magnitude higher in value at the end point of the pathway. This difference in magnitude and the stark variance in global secondary structure again indicates that different aggregation pathways are occurring for the αS variants, but nonetheless that the peptides are effective in modulating aggregation, although their mechanism of action may vary.

### The Distribution of Oligomer Species Varies between αS Variants and Is Modulated by the PCA-Peptides

In order to investigate if the peptides vary the distribution of oligomer species formed, samples from the ThT end-point (Figure 4) were analyzed using PICUP.^46^ In PICUP, neighboring protein side chains can form an intermolecular cross-link through a radical mechanism.^47,48^ Through this mechanism, PICUP can rapidly and efficiently form covalent bonds to stabilize protein assemblies, without the requirement for unnatural structural modifications to the protein.

**Figure 6:**
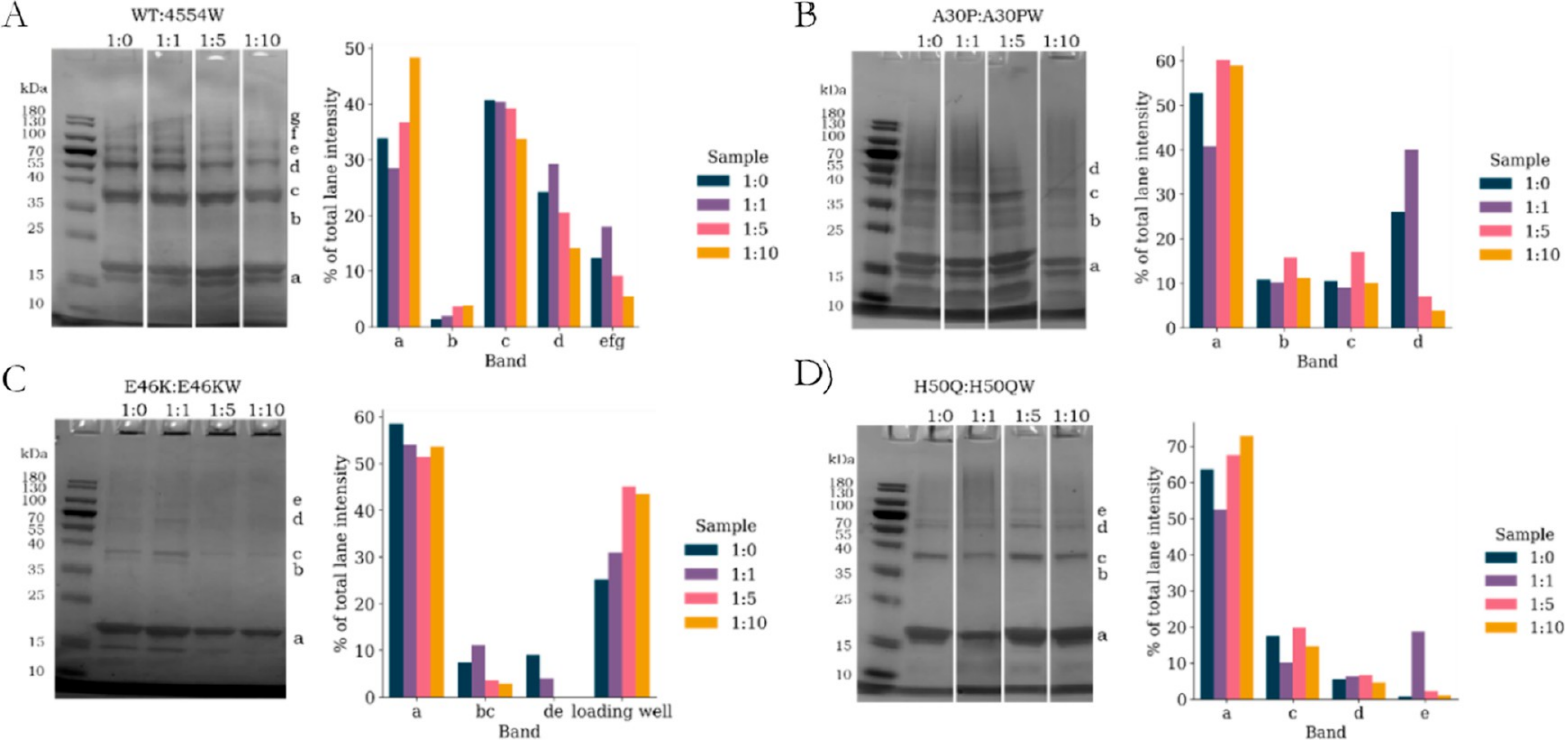
Oligomer formation analyzed by PICUP and SDS_PAGE. A) WT + 4554W, B) A30P+A30PW, C) E46K+E46KW and D) H50Q + H50QW. For each sample: Left) SDS-PAGE of dose-response of αS+peptide (where 1:0 refers to the αS control) following PICUP. The band labels correspond to monomer (∼15 kDa) (a) and a range of oligomer species ranging from dimer to octamer [(b) ~ 30 kDa, (c) ∼37 kDa, (d) ∼60 kDa, (e) ∼75 kDa, (f) ∼90 kDa and (g) ∼120 kDa]. Right) Band intensities [analysed in ImageJ (Fiji)] of the SDS-PAGE bands as a percentage of sum of the total intensity of each lane; lane efg refers to the combined values for the largest oligomers formed.

In Figure 6, the oligomer distribution of αS variants with their respective PCA peptides are summarized. In all cases, except H50Q, the increase in peptide concentration resulted in a decrease in the concentration of higher order oligomers [bands d-g, ∼60 kDa (possible tetramer) to ∼120 kDa (possible octamer)]. However, slightly different distribution patterns were observed for several of the αS variants.

In the case of WT and 4554W (Figure 6A), in addition to the decrease in the higher order oligomer bands, there was a slight increase in intensity measured for the b band at ∼30 kDa, possibly representing a peptide-stabilized dimer. With regards to E46K (Figure 6C), a decrease in all of the oligomer bands [including c band (possible dimer or trimer) which remains mostly constant for the other αS variants] was observed with increasing concentrations of E46KW. This decrease in oligomer concentration had a corresponding increase in the sample trapped in the loading well, indicating that the species formed were particularly large. This may also correspond to the large ThT values and significant β-sheet CD spectra obtained. Similar to the other αS variants, the concentration of A30PW did not significantly alter the intensity of the c (∼37 kDa, possible dimer or trimer) band (Figure 6B). However, unlike the other αS variants, the b band (possible dimer) remained prominent for all samples and was also not affected significantly by A30PW, indicating that A30PW does not vary the distribution of these lower-order oligomers. H50Q (Figure 6D) was the only αS variant analyzed by PICUP, where there was not a reduction in the higher-order oligomer bands, and on the contrary, an increase in the intensity of the e band (∼105 kDa, possible heptamer) was observed and was particularly prominent for the lowest H50QW concentration.

### Characterization of Peptide Winners against All Targets

**Figure 7:**
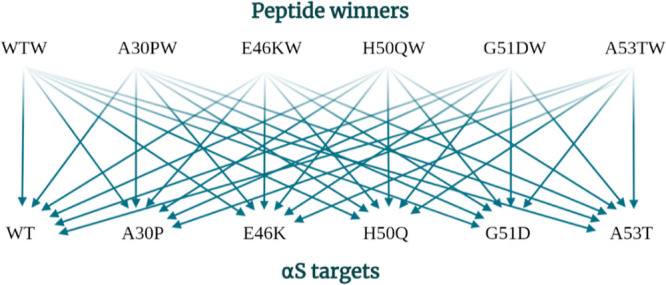
To develop a more stringent assay, the effectiveness of each peptide winner (WTW, A30PW, E46KW, H50QW, G51DW, and A53TW) in modulating the aggregation of each αS target (WT, A30P, E46K, H50Q, G51D, and A53T) was investigated.

In order to develop a more stringent assay to identify a peptide that has potential for further development as a therapeutic effective against WT (affecting most people with PD) and also one or more of the more toxic forms associated with early-onset PD, we expand on the experiments described in the previous sections to determine if any of the peptides are effective in modulating the aggregation of multiple αS variants (Figure 7).

### The Peptides Modulate Aggregation of Their Respective and Alternative Targets

To determine the ThT aggregation kinetics, we again employed a lipid-induced primary nucleation method. Specifically, each monomeric αS variant (100 μM) was incubated with each PCA peptide winner (100–1000 μM) and DMPS SUVs (200 μM) at 30 °C under quiescent conditions. We also compared the newly identified peptides to our previously optimized version of 4554W–4554W(N6A)^49^—as a control and benchmark for future developments.

**Figure 8:**
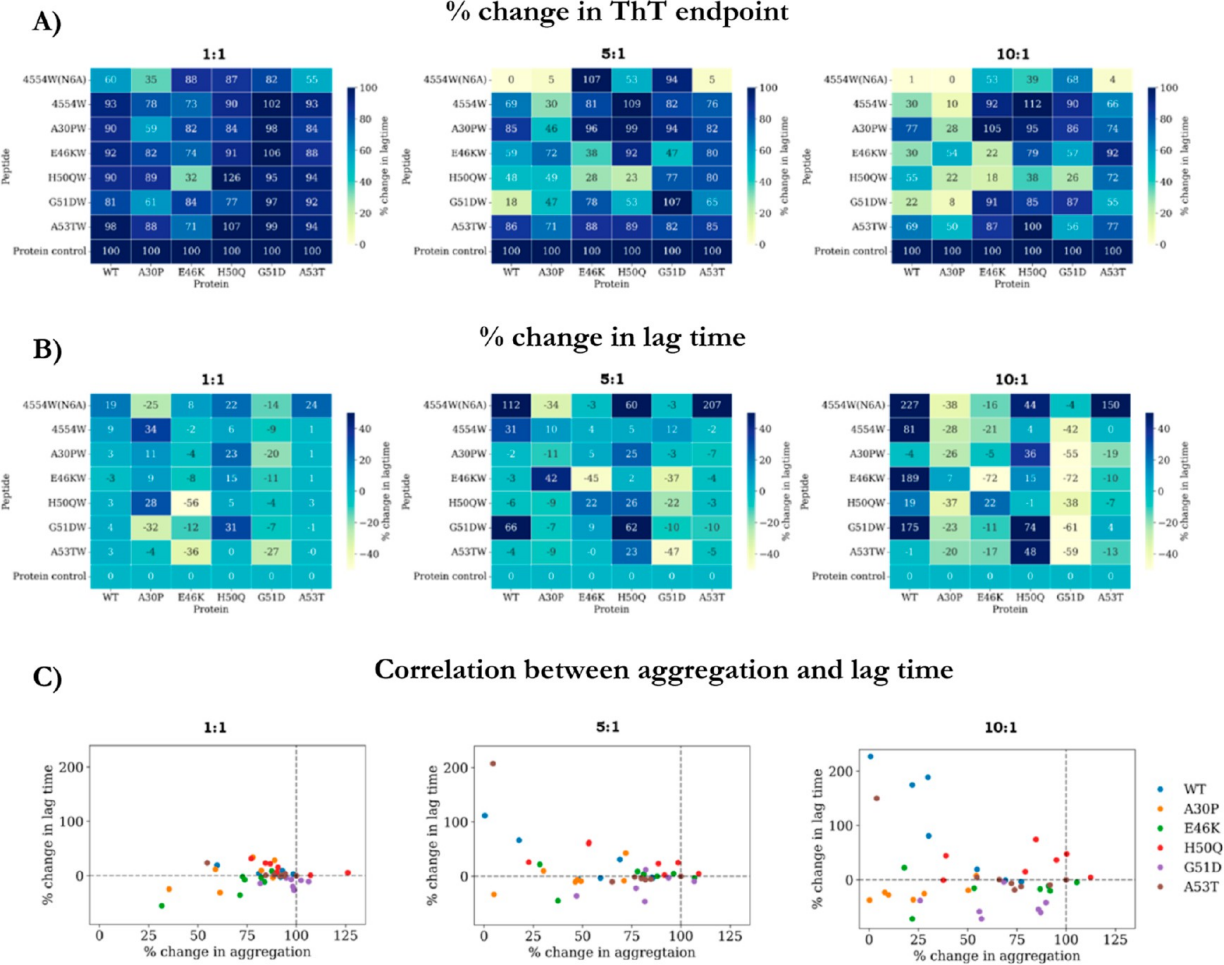
Summary of ThT result for all αS variants incubated with all PCA peptides. (A) ThT end point intensity for each sample as a percentage of the respective αS variant control. (B) Percentage change in lag time (determined via midpoint change, described in Methods), with negative values representing shorter lag times and positive values representing increased lag times. (C) Change in aggregation (ThT end point intensity) vs change in lag time was plotted as a scatter plot to determine any correlation between these two parameters was observed. The colored dots represent each αS variant [WT (blue), A30P (orange), E46K (green), H50Q (red), G51D (purple), and A53T (brown)], and the version showing the trend for each peptide can be found in Figure S2. Full ThT profiles are shown in Figure S1.

As seen in the previous section, the vast majority of the peptides result in a reduction in ThT intensity (Figure 8A) at the end point of the aggregation assay (full ThT results are shown in Figure S1). Inhibition is most prominent for WT and A30P, where all of the peptides (at a 1:10 peptide excess) resulted in substantial reduction in the ThT intensity. Conversely, overall, the peptides are least effective in reducing the ThT intensity of H50Q and G51D, where on average the peptides only reduced the ThT intensity by ∼20%.

In addition to reducing ThT intensity, the peptides also significantly reduced the lag time of some of the variants (Figure 8B), in particular G51D. This strongly indicates that a different mechanism of aggregation is occurring. The relationship between lag time and aggregation was explored, and trends were analyzed by scatter plots (Figure 8C). The individual αS variants showed some trends in their response to the peptides, for example, G51D (purple) aggregation is found to be modulated mostly through reducing lag time and a modest reduction in ThT intensity, whereas H50Q (red) shows a similar modest reduction in ThT intensity but an opposite effect in the lag time modulation. The scatter plot showing the trends of the peptides is shown in Figure S2, where it can been seen that the most dramatic effects in αS modulation occur with 4554W(N6A), and these effects are also prominent at a 1:5 ratio and not only at 1:10 where most of the other peptides are most effective.

Surprisingly, we observed that each PCA peptide is not necessarily the most effective peptide inhibitor for its respective target. For example, for A53T, the most effective peptide inhibitor is 4554W(N6A), followed by G51DW, according to the ThT and CD data. The A53TW peptide in this case is overall the worst-performing peptide, as measured by ThT-active aggregation. The PCA-derived peptide is selected due to being a strong binder, resulting in a subsequent reduction in the toxicity. Therefore, this could suggest that the PCA assay is either not selecting the best peptide inhibitor or that these ThT aggregation assays do not necessarily correlate to in vivo toxicity. For example, the formation of (ThT-active) fibrils could be a mechanism to remove oligomer species and hence reduce in vivo toxicity. Consequently, samples that result in no increase in the ThT signal could be prolonging or stabilizing the presence of oligomer species that may or may not be more toxic than the monomer and/or fibrils. As a result, we have used further methods to characterize the peptides to understand more about how they modulate the αS structure and function, including CD (Figure S4), to understand more about the modulation of the global secondary structure (with the understanding that structures with a high β-sheet content result in increased toxicity), TEM to visualize the formation of oligomers or fibrils in the presence of the peptides, and PICUP to quantify the range and abundance of a range of oligomer species.

### Despite the Aggregation Assays Showing a Significant Reduction in ThT-Active Fibrils, Fibrils Were Still Observed Via TEM

Visualizing fibril formation at the lipid-induced aggregation end point provides a method to determine if the ThT assay results correspond to changes to the fibril load formation or a change in the fibril morphologies observed. In particular, the peptides corresponding to the greatest reduction in ThT intensity for each αS variant were investigated and imaged.

**Figure 9:**
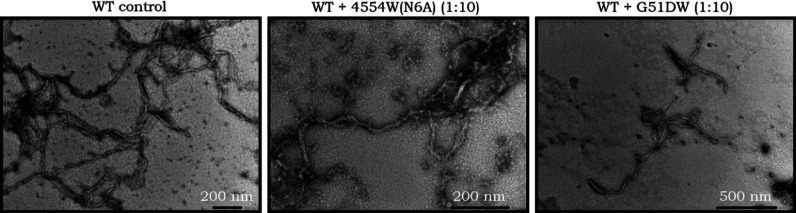
TEM of WT with 4554W(N6A) and G51DW at a 10-fold excess. End-point samples from the lipid-induced primary nucleation ThT assays show that the WT control formed fibrils under these conditions. In the presence of 4554W(N6A) (10-fold excess), the prevalence and maturity of the fibrils decreased, with a significant number of oligomer-type structures also visualized. With G51DW (10-fold excess), the fibrils observed were much shorter and the prevalence was less than that of the WT control.

In the case of WT, A30P and A53T, the most effective peptides, determined via ThT aggregation assays, were found to be 4554W(N6A), followed by G51DW. TEM images of the WT control (100 μM), WT + 4554W(N6A) (1:10), and WT + G51DW (1:10) were compared (Figure 9). Both of these apparently effective peptides substantially reduced the formation of fibrils. However, differences between the samples were observed such as 4554W(N6A) had some fibril formation but with several oligomer species present, whereas fewer oligomers were observed with G51DW and the fibrils were shorter. This potentially corresponds to the slightly higher ThT value obtained for G51DW, and the oligomers formed with 4554W(N6A) may not be ThT-active.

**Figure 10:**
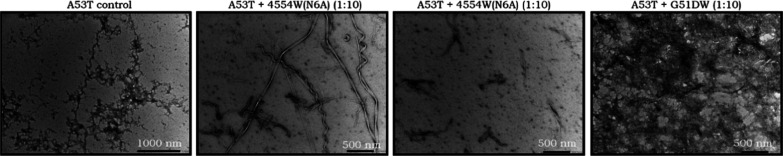
TEM of A53T with 4554W(N6A) and G51DW at a 10-fold excess. End-point samples from the lipid-induced primary nucleation ThT assays show that the A53T control formed fibrils under these conditions. However, in the presence of 4554W(N6A) (10-fold excess), in contrast to the A53T control, helical polymorphs were observed although a significant number of protofibril-type structures were also visualized. With G51DW (10-fold excess), no fibrils or any features with distinct morphologies were visualized.

TEM images from A53T control, A53T + 4554W(N6A) (1:10), and A53T + G51DW (1:10) were also compared (Figure 10). Under these conditions, we did not observe any helical fibril formation for the A53T control. However, once incubated with 4554W(N6A), helical polymorphs were visualized, indicating that 4554W(N6A) may be able to accelerate the formation of these mature helical fibrils. Notably, 4554W(N6A) did not exclusively form helical fibrils but also formed protofibril-type fibrils. On the other hand, when incubated with G51DW, no fibrils were observed, nor were there any distinct morphological features. This indicates that these peptides may have a divergent effect on the aggregation of A53T. This result is somewhat surprising, given that G51DW resulted in a greater ThT intensity than 4554W(N6A) (Figure S1), and the CD spectra obtained are also similar between the two samples (Figure S4).

**Figure 11:**
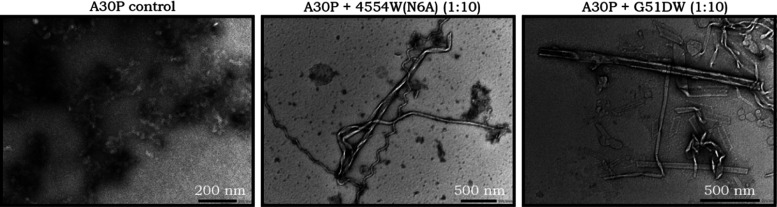
TEM of A30P and 4554(N6A) and G51DW at a 10-fold excess. End-point samples from the lipid-induced primary nucleation ThT assays show that the A30P control does not form mature fibrils under these conditions, instead the prominent species were protofibrils. However, in the presence of both 4554W(N6A) and G51DW (10-fold excess), various mature fibril morphologies were observed.

The fibril formation of A30P (Figure 11) differs significantly from WT and A53T in that no mature fibrils are observed under these conditions and timeframe, with the prominent species being protofibrils. However, when incubated with both 4554W(N6A) and G51DW, fibrils were present, indicating again that the peptides may be able to accelerate the formation of fibrils for some of these αS mutants.

**Figure 12:**
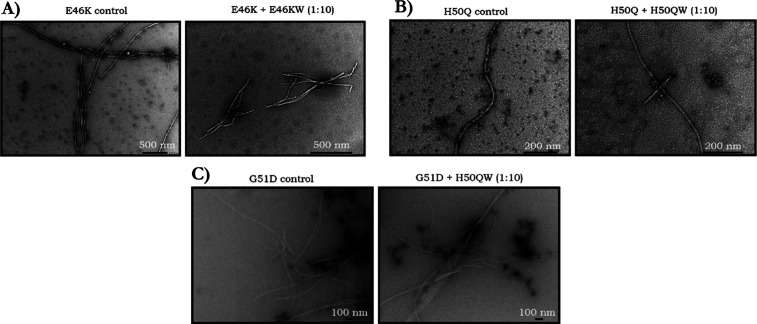
TEM of E46K, H50Q, and G51D with their best-performing peptide inhibitors. (A) End-point samples from the lipid-induced primary nucleation ThT assays show that the E46K control forms mature fibrils under these conditions, and while fibrils were still observed with E46KW (10x excess), these were significantly shorter. (B) End-point samples from the lipid-induced primary nucleation ThT assays show that the H50Q control forms mature fibrils under these conditions, and while fibrils were still observed with H50QW (10x excess), additional shorter and thicker fibrils were also visualized. (C) End-point samples from the lipid-induced primary nucleation ThT assays show that the G51D control forms mature fibrils under these conditions, and despite resulting in the lowest ThT intensity and a reduction in β-sheet character, when incubated with H50QW, similar fibril morphologies and abundance were observed.

For the remaining three αS variants—E46K, H50Q, and G51D—different most effective peptides were obtained. In Figure 12, example TEM images for the E46K control compared to the E46K + E46KW (1:10) samples show that when incubated under these conditions, E46K is still able to form fibrils when incubated with E46KW (10× excess). However, the fibrils observed were generally shorter and the abundance was lower. A similar trend was observed with H50Q + H50QW, where for the H50Q control, long, thin meandering-type fibrils were the predominant species observed, and while these continued to be observed with H50QW, a number of shorter, thicker, and more interesting types of fibrils were observed. Therefore, from these observations, despite the significantly reduced ThT intensity and reduction in β-sheet formation (Figure S4), fibril formation is not eradicated with these peptides against these variants under these conditions; however, it would appear that the fibril formation has been modulated in a variety of ways. The biological significance of this can be investigated further through cell-based assays, which are briefly explored in the next section.

### Do Peptides Protect against αS-Induced Toxicity in a Cell-Based MTT Assay?

To determine the effect the peptide winners have on modulating the cytotoxicity resulting from αS, an assay was developed using cultured human neuroblastoma SH-SY5Y cells. This assay involved aging αS (100 μM) in the presence of each of the peptide winners (100 μM, 1 equiv) at 30 °C for 24 h under quiescent conditions. Following the incubation period, the αS-peptide mixture was added to differentiated SH-SY5Y cells at a final αS concentration of 20 μM, and following further incubation at 37 °C for 48 h, cell viability was determined using an MTT assay.

**Figure 13:**
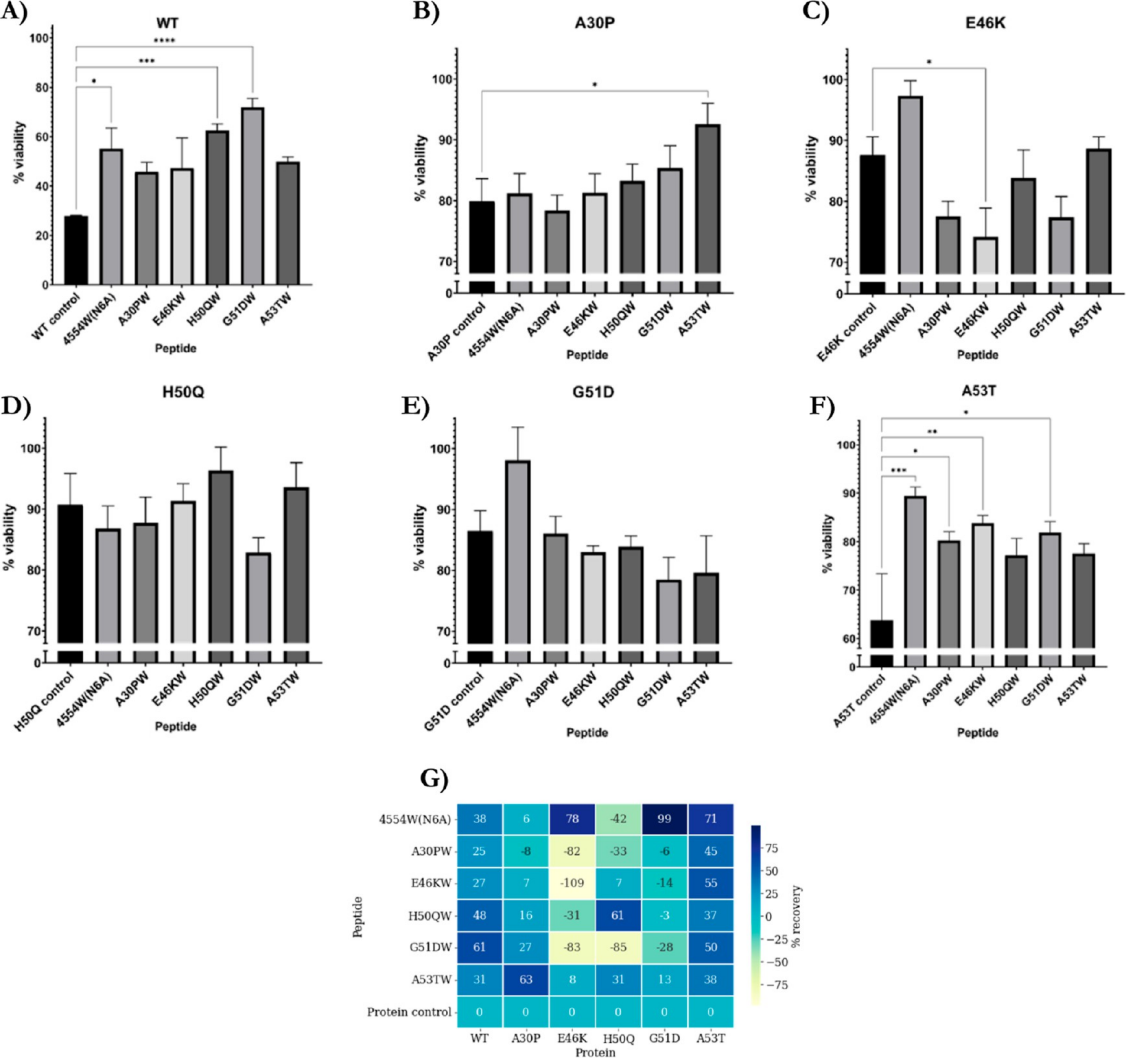
MTT results for peptide recovery from αS-mediated toxicity. (A–F) Results for WT, A30P, E46K, H50Q, G51D, and A53T, respectively. αS samples of the respective variants (100 μM) were incubated with the peptide winner samples (100 μM, 1:1) at 30 °C under quiescent conditions of 24 h % viability are normalized to the respective buffer solutions, representing the 100% value. The results are the mean of three independent repeats ± SEM. *p < 0.05, **p < 0.01, ***p < 0.001, and ****p < 0.0001; two-way ANOVA followed by Tukey’s multiple comparisons test. (G) Percentage recovery of all peptides and αS variants was calculated by determining the toxicity window for each αS mutant followed by the peptide recovery.

Peptides were able to protect against αS-mediated toxicity of differentiated SH-SY5Y cells to varying extents (Figure 13). In particular, protection from αS-mediated toxicity under these conditions was most notable for A53T (recovery ranging from 37 to 71%) and WT (ranging from 25 to 61%), while five out of the six peptides protected to some extent against A30P-mediated toxicity (range from 6 to 63%); however, A30PW showed an 8% reduction in cell viability. Very few peptides were able to protect against E46K- and G51D-mediated toxicity and most enhanced toxicity, with the respective peptide winners (E46KW and G51DW) being the worst-performing. However, in both cases, A53TW and 4554W(N6A) were protective, with 4554W(N6A) demonstrating greater rates of protection with 78 and 99%, respectively. In the case of H50Q, three peptides resulted in protection from modest αS-mediated toxicity, and in this case, H50QW resulted in the most significant extent of protection with a calculated value of 61%. Out of all the αS-peptide interactions reported herein, this is the only pairing where the respective peptide winner protects αS-mediated cell-toxicity the most.

Furthermore, we are able to see that the peptides are able to protect against toxicity as a result of all of the αS variants, not only WT. These results are consistent with the other results described herein: the peptides work through inhibiting the earliest stages of αS aggregation, in particular, these MTT results highlight that they are able to reduce the production of αS toxic oligomers, the presumed disease-relevant species, that result in neuron death.

### Analysis of the Structure–Activity Relationship

**Figure 14:**
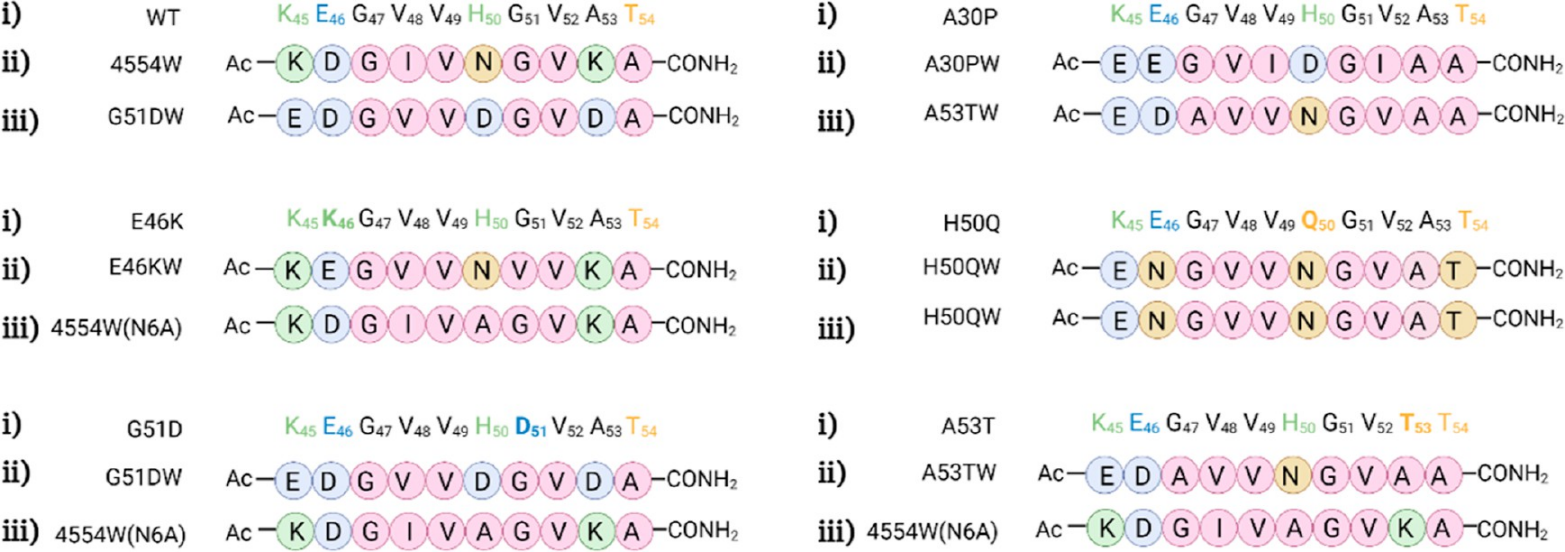
Summary of peptide results. (i) Sequence of respective αS targets. Point mutations are in bold, green represents positively charged, blue represents negatively charged, and orange represents polar amino acid. (ii) Peptide sequence derived from the PCA screen against the respective αS variant. (iii) Best-performing peptide, as determined by MTT cell toxicity.

In addition to evaluating the effectiveness of the peptides, another aim of this work was to determine if a greater understanding about the residues required for αS inhibition could be gained. Through evaluating the peptide sequences generated from the library screen (Figure 14), it was shown that there was a high conservation of the WT residues at positions 3, 4, 5, 7, and 8 (although no one position selected the same residue for all of the hits), which were all hydrophobic, and the smallest residue was selected in the majority of cases. The most divergence from the wild-type sequence was observed for positions 1, 6, and 10. From our previous work, alanine at the 6 position resulted in a much improved version of 4554W [e.g., 4554W(N6A)],^49^ indicating that modifying these peptides with an additional hydrophobic residue would be advantageous. Moreover, our previous work also showed that the loss of K1 did not result in a loss of efficacy, and this is further supported here by the fact that the screen flips between selecting K and E. Interestingly, the mutation selections (e.g., 46K) were never selected from any of the screens, despite the high conservation of the WT residues.

Through evaluation of the ThT results, overall 4554W(N6A) and G51DW were most consistent in reducing the ThT intensity values for all αS variants. However, in the cell toxicity assays, where 4554W(N6A) excelled in recovering toxicity, G51DW had the opposite effect and appeared to enhance toxicity for a number of the αS variants. Additionally, H50QW was often found to have a high level of inhibition, and this corresponded well to the conservation of random coil structure in CD in many cases. Furthermore, the toxicity recovery was good in most cases too, however, it was also the most challenging peptide to handle due to poor solubility making purification challenging, with a tendency to aggregate, which can mostly be explained through the fewer charged residues in the sequence.

## Conclusions

Using a semi-rational intracellular PCA library screen, we have previously identified the peptide 4554W, which was shown to function by inhibiting lipid-induced primary nucleation.^34,35^ Despite the library being designed based on the familial mutations associated with early-onset PD, this library had not been screened against the αS variants. Here, we build upon our previous work to describe the identification and characterization of five peptides identified from library screens against five of the known αS variants associated with early-onset PD, with the aim to identify a peptide that is effective against both WT and the αS variants associated with early-onset disease.

The peptides were characterized through a range of methods including ThT aggregation assays, CD, cross-linking, and cell toxicity assays. The five peptides identified all reduced aggregation of the respective target, with several also identified to be effective at reducing aggregation of the other variants. Interestingly, the PCA winner peptide was not always the most effective peptide for each respective αS variant. This may be explained by the wide variety of methods in which the peptides can bind and reduce toxicity, and not necessarily a failure of the PCA screen. For example, the difference in efficacy could be due to a preference in aggregation conditions for inhibition not explored herein, such as elongation or secondary nucleation, or traditional-style shaking assays may be a more appropriate method of evaluation in some cases. Nonetheless, each peptide exhibited some activity in a dose–response manner, and thus, the PCA screen can be deemed a success.

Our results have shown that our previously optimized peptide 4554W(N6A) is highly effective against not only WT αS but also several of the variants associated with early-onset PD and hence is a suitable baseline for further work toward a therapeutic for PD. Moreover, a greater understanding of the structure–activity relationship was gained through evaluation of the peptide performance. From this, a hydrophobic core of the peptide appears essential for improved activity, but charged residues at both the N- and C-terminus improve activity, along with enabling solubility. Along with 4554W(N6A), G51DW showed promising activity for future development. Therefore, aspects of both of these peptides should be considered when looking to develop the peptides further, for example, through peptidomimetics or cyclization to develop a more drug-like peptide.

These peptides have potential to not only be developed as therapeutics but also through modification of their structures—for example, through the addition of a fluorophore—there is potential for them to be developed as probes for a potential diagnostics application tool or as theranostics.

## Methods

### αS Expression and Purification

Full-length (1–140) α-synuclein (αS) was recombinantly expressed in BL21(DE3) *Escherichia coli* cells using a pET21a plasmid (WT αS plasmid was a gift from the Michael J. Fox Foundation, Addgene plasmid # 51486) and purified based on previously published methods. Subsequent early onset mutants were created using site-directed mutagenesis using the wild-type gene.

Briefly, overnight cultures (2xYT, 10 mL) of the transformed *E. coli* were used to inoculate 2xYT (1 L) cultures containing ampicillin (100 mgL^–1^), which were grown (37 °C, 200 rpm) to an OD_600_ of 0.6–0.8. Protein expression was induced with IPTG (final concentration of 1 mM), and cells were harvested by centrifugation (5000 rpm, 20 min, 4 °C) following incubation (37 °C, 200 rpm, 4 h). The bacterial cell pellet was resuspended in 20 mM Tris buffer (pH 8) with a 1 cOmplete protease inhibitor tablet (Roche). Following freeze-thawing at −20 °C, the cells were lysed by sonication. The soluble fraction of the lysate was separated from the cell debris by centrifugation (20 000 rpm, 20 min, 4 °C) and boiled (95 °C, 10 min) to precipitate impurities (αS remains soluble). Precipitated proteins were discarded after centrifugation (18 500*g*, 20 min, 4 °C), and ammonium sulfate was added to the supernatant to create a 30% solution, which was gently agitated (RT, 1 h) to precipitate the protein (including αS). The precipitated protein was collected by centrifugation (18 500 *g*, 20 min, 4 °C) and resuspended with gentle agitation in 20 mM Tris buffer (pH 8, 4 °C). The protein was purified by anionic exchange chromatography on an ÄKTA pure purification system (GE Healthcare) with a 5 mL HiTrap Q HP (GE Healthcare) pre-packed column. Fractions containing the purified protein were combined and further purified by size exclusion chromatography using a HiLoad 16/60 Superdex 75 pg (GE Healthcare) pre-packed column and buffer-exchanged into the experimental buffer (20 mM sodium phosphate buffer (pH 6.5)). αS eluted between 54 and 64 mL. The protein was aliquoted and flash frozen in liquid nitrogen and stored at −80 °C until required.

The concentration of purified αS was determined by UV (280 nm) using a 2 mm quartz cuvette and an excitation coefficient (ε) of 4836 M^–1^ cm^–1^. Purity of the eluted protein was confirmed by SDS-PAGE, and the correct product was confirmed via mass spectroscopy using an Agilent QTOF (ESI-QTOF) mass spectrometer. CD spectral scan was used to confirm that the monomeric stock solutions of αS were random coil (Figures S5–S10).

### Peptide Synthesis and Purification

Peptides were synthesized on a H-Rink Amide Chem matrix resin (0.22 g, 0.1 mmol) using a Liberty Blue microwave peptide synthesizer (CEM) and standard techniques of Fmoc SPPS.

Coupling of Fmoc-protected amino acids was achieved via double-coupling using Fmoc-protected amino acid (1 mmol), (benzotriazol-1-yloxy)tripyrrolidinophosphonium hexafluorophosphate [0.5 M in dimethylformamide (DMF)], and *N*,*N*-diisopropylethylamine (17% in DMF (v/v)). Following coupling, piperidine (20% in DMF, with 5% formic acid) was used to deprotect the Fmoc-protecting group. 5% formic acid was required in order to minimize aspartimide formation.^50^ The peptides were acetylated using acetic anhydride (20% in DMF).

Following synthesis, the peptides were cleaved from the resin with simultaneous removal of the side-chain protecting groups through addition of a cleavage solution [10 mL: trifluoroacetyl (TFA) (95%), triisopropylsilane (2.5%), and H_2_O (2.5%)] for 3.5 h at RT. The cleaved resin was removed by filtration, and the filtrate was precipitated into ice-cold diethyl ether. The peptide pellet was obtained via vortexing, followed by centrifugation (7000 rcf, 10 min, 4 °C). This method was repeated three times with ice-cold diethyl ether added to the crude peptide pellet.

The resulting crude peptide was dissolved in high-performance liquid chromatography (HPLC) buffer (5% ACN, 95% H_2_O). Purification was performed using a preparative scale reverse phase HPLC using a Phenomenex Jupiter Proteo reverse-phase column (4 μm, 90 Å, 250 × 21.2 mm), using eluents A (H_2_O with 0.1% TFA) and B (ACN with 0.1% TFA). Collected fractions were analyzed by electrospray mass spectrometry (ESI). Those found to contain the desired product were pooled and lyophilized. The purified pellet was stored at −80 °C.

### Preparation of Lipid (DMPS) Vesicles

Suspension of 1,2-dimyristoyl-*sn*-glycero-3-phospho-l-serine (sodium salt) (DMPS) in 20 mM sodium phosphate buffer (pH 6.5, 2 mM) was incubated on a Thermomixer compact (Eppendorf) shaker (45 °C, 1400 rpm, 3 h). The solution was frozen and thawed five times using dry ice (15 min) and a Thermomixer compact (Eppendorf) shaker at 45 °C (0 rpm, 5 min). Lipid vesicles of the desired size were formed via sonication (Soniprep 150 plus sonicator, amplitude 10, 5 × 30 s with 30 s rest between rounds). Vesicle size distribution was measured using dynamic light scattering using a Zetasizer Nano ZSP (Malvern Instruments) to ensure that a final consistent size of between 30 and 40 nm was obtained. The lipid concentration used is the monomer equivalent concentration.

### Measurement of Aggregation Kinetics Using Lipid Vesicles

Solutions containing αS (100 μM), DMPS vesicles (200 μM), ThT (50 μM), and sodium azide (0.01%) along with peptide winners (100, 500, or 1000 μM) in 20 mM sodium phosphate buffer (pH 6.5) were prepared in a half-area 96-well nonbinding plate (Corning 3881) or 384-well nonbinding plate (Corning 3766), sealed with aluminum Thermowell sealing tape (Corning 6570), and incubated in a CLARIOstar plate reader (BMG Labtech) for up to 400 h at 30 °C under quiescent conditions. Samples had a volume of 100 μL (96-well) or 50 μL (384-well). Readings were taken at 1200 s intervals; λex = 440–10 nm and λem = 480–10 nm, gain = 800, focal height = 4.9 mm. Each experiment was carried out in triplicate, and error bars represent the standard error.

Aggregation kinetics analysis: percentage change in aggregation was calculated for each sample by normalizing to the αS variant control samples (100%). The percentage change in lag time was calculated by through the change in the mid-point time, determined via fitting each of the sigmoidal curve to the equation
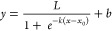
where *L* is the maximum intensity value of the curve (e.g., *y*_max_), *b* is the minimum value of the curve (e.g., *y*_min_), *k* is the logistic growth rate, and *x*_0_ is the curve midpoint.

### Transmission Electron Microscopy

αS samples from the end-point of the aggregation assays were collected. 5 μL of these samples was put onto on glow-discharged Formvar/carbon-coated, 200 mesh, copper grids for 1 min. The samples were dried with a filter paper, washed twice with MilliQ water for 1 s, and removed each time with a filter paper. The sample was stained by incubating the grids with 5 μL of Uranyl Acetate Zero (Agar Scientific) for 30 s, followed by removal of the excess stain with a filter paper. The grids were left to air-dry for 2 h. The samples were imaged using a TEM Jeol 2100 Plus (JEOL) operating at an accelerating voltage of 200 kV. Multiple grids were screened to obtain representative images of the samples.

### CD Spectroscopy

Far-UV CD spectra were recorded using a Chirascan V100 (Applied Photophysics) with a Peltier thermally controlled cuvette holder. Quartz cuvettes with a 1 mm pathlength were used, and CD spectra were obtained by averaging three individual spectra recorded with a 1 nm bandwidth, ranging between 280 and 190 nm. Each sample had a final αS concentration of 10 μM and was blanked against the buffer used in the aggregation assay.

Data are plotted as the mean residue ellipticity (MRE), which was calculated using the following equation
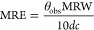
where MRE is the mean residue ellipticity (deg cm^2^ dmol^–1^), θ_obs_ is the observed ellipticity (mdeg), MRW is the mean residue weight (molecular mass/(N^–1^), where N is the number of amino acids, for αS this is ∼104), d is the pathlength (cm), and c is the concentration (mg/mL).

### Photo-Induced Cross-Linking of Unmodified Proteins

To the αS aggregation end-point samples (20 μL, 100 μM), tris(2,20bipyridyl)dichloro-ruthenium(II) hexahydrate (Ru(bpy) (2 μL, 1 M in 20 mM sodium phosphate buffer pH 6.5) and 20 mM ammonium persulphate (2 μL, 20 mM in 20 mM sodium phosphate buffer pH 6.5) were added. The samples were irradiated with visible light (20 s) and the reaction quenched with dithiothreitol (2 μL, 1 M in water), followed by addition of SDS-PAGE sample buffer [RunBlue LDS sample buffer (4× concentrate) (Expedeon)]. The distribution of oligomers was determined using SDS-PAGE and Coomassie staining. Briefly, 10 μL of each cross-linked sample was electrophoresed on a 12% tricine gel (Expedeon) using RunBlue run buffer and visualized using InstantBlue Coomassie stain.

The gel band intensities were analyzed using ImageJ (Fiji). The image was converted to 32-bit, and the band intensities were determined using the Gel Analyser tool. Data were exported to Excel, and the relative percentage of the band intensities for each lane were calculated.

### Neuroblastoma Cell Culture

Human neuroblastoma cell line SH-SY5Y (ECACC 94030304) was purchased from Public Health England’s European Collection of Authenticated Cell Cultures (ECACC). Unless otherwise stated, all cell culture consumables were purchased from ThermoFisher. Cells were cultured in Dulbecco’s modified Eagle’s medium (DMEM)/F-12 media with phenol red and without *N*-(2-hydroxyethyl)piperazine-*N*′-ethanesulfonic acid and l-glutamine. DMEM/F12 was supplemented with fetal bovine serum (10%), l-glutamine (2 mM), and non-essential amino acids (5%); with penicillin (100 IU) and streptomycin (100 mg/mL) (Corning). The culture was maintained in an incubator at 37 °C, 5% CO_2_, and saturated humidity until about 80% confluency was reached for a maximum of 20 passages. For toxicity assays, the stock culture was seeded in 24-well plates and grown at 37 °C, 5% CO_2_, and saturated humidity for 24 h to reach 60% confluency prior to differentiation. Cells were seeded at 1 × 10^6^ cells/mL.

### SH-SY5Y Differentiation

The differentiation of SH-SY5Y cells was carried out based on the method from Förster et al. using two steps and phase 1 and phase 2 media.^51^ The phase 1 medium (DMEM containing l-glutamine (4 mM) and glucose (25 mM), P/S (1%) and no sodium pyruvate; retinoic acid (10 μM, Merck) was added just prior to addition to cells) was added to the cells on DIV 1. The phase 2 medium (Neurobasal A medium without phenol red, l-glutamine (1%), N-2 supplement (1%), and P/S (1%); human brain-derived neurotrophic factor (50 ng/mL, Merck) was added shortly before adding to the cells) was added to the cells on DIV 5. Cells were left to differentiate until DIV 8 at 37 °C, 5% CO_2_, and saturated humidity.

### Cell Toxicity assays (MTT)

Aliquots of the incubated samples were added to the media of differentiated SH-SY5Y neuroblastoma cell cultures to a final concentration of 20 μM αS in triplicate. The plate was incubated for 48 h at 37 °C, 5% CO_2_, and saturated humidity. Cell viability was assessed by a 3-(4,5-dimethylthiazol-2-yl)-2,5-diphenyltetrazolium bromide (MTT) (Invitrogen) reduction assay. Briefly, the cell growth media was removed and replaced with an equivalent volume of growth media containing 1 mg/mL of MTT solution at 37 °C, 5% CO_2_, and saturated humidity for 1 h. The MTT solution was then removed, and the resulting blue formazan was resuspended in 150 μL of 2-propanol. The absorbance of the blue formazan solution was measured at 595 nm and presented as an average of the three wells for each condition. Data were blanked against wells treated with hydrogen peroxide (1 mM) to represent 100% toxicity.
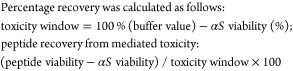


## Supporting Information Available

Full ThT aggregation profiles for each αS variant vs each PCA peptide; correlation between lag time and aggregation for each peptide; MTT of the peptide controls; CD of aggregation end point for each αS variant vs each PCA peptide; and αS purification overview (anionic exchange and size exclusion chromatograms, SDS-PAGE, CD, and mass spectroscopy) (PDF)
