# Supplementary material for: Library-Derived Peptide Aggregation Modulators of Parkinson’s Disease Early-Onset α-Synuclein Variants
Source: ACS Chem Neurosci. 2022 May 25;13(12):1790–804. doi: 10.1021/acschemneuro.2c00190 (PMC9204772; doi:10.1021/acschemneuro.2c00190)
Supplement: Supplementary file 1 — cn2c00190_si_001.pdf [file cn2c00190_si_001.pdf]

Supporting Information

Library-derived peptide aggregation modulators of Parkinson's disease early-onset α-synuclein variants.

Kathryn J. C. Watt, Richard M. Meade, Robert J. Williams, and Jody M. Mason*

Depart of Biology and Biochemistry, University of Bath, Claverton Down, BA2 7AY, United Kingdom

**To who correspondence should be addressed:* j.mason@bath.ac.uk

Keywords: peptides, amyloid aggregation, early onset Parkinson’s disease

Full ThT lipid-induced aggregation pathway for all αS variants and PCA-peptides

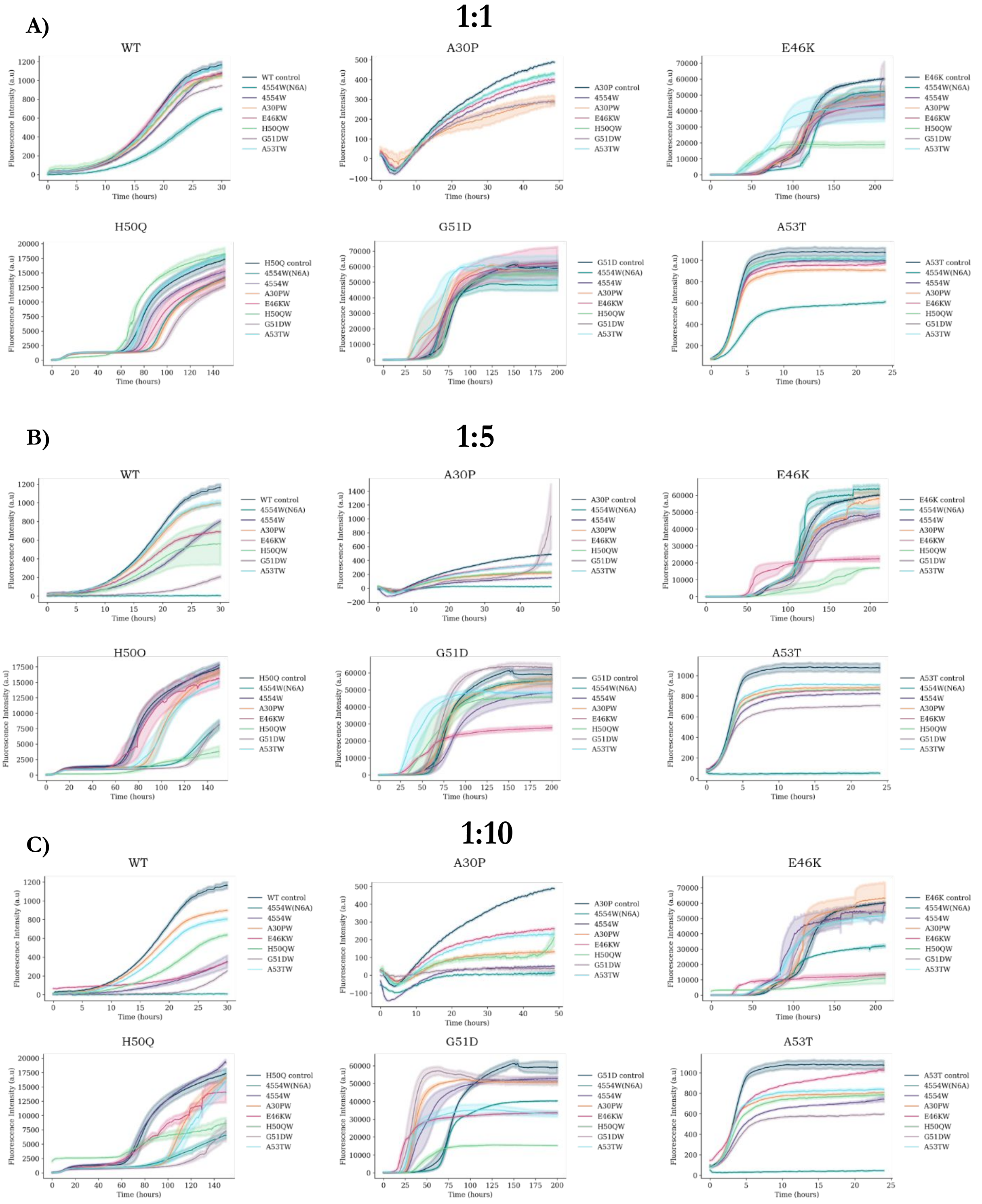

Figure S1: ThT lipid-induced aggregation results for each αS variant and each PCA-peptide. Each αS variant (100 µM (dark blue)) was incubated with each PCA winner peptide (4554W(N6A) (teal), 4554W (purple), A30PW (orange), E46KW (pink), H50QW (green), G51DW (mauve), or A53TW (light blue)), DMPS SUVs (200 µM) and ThT (50 µM) in 20 mM sodium phosphate buffer, pH 6.5 at 30 °C under quiescent conditions until the ThT signal plateaued (up to 250 hr). The average of three repeats is shown with the standard error. **A)** αS variant: peptide ratio is 1:1 (e.g 100 µM αS and 100 µM peptide), **B)** αS variant: peptide ratio is 1:5 (e.g 100 µM αS and 500 µM peptide) and **C)** αS variant: peptide ratio is 1:10 (e.g 100 µM αS and 1000 µM peptide).

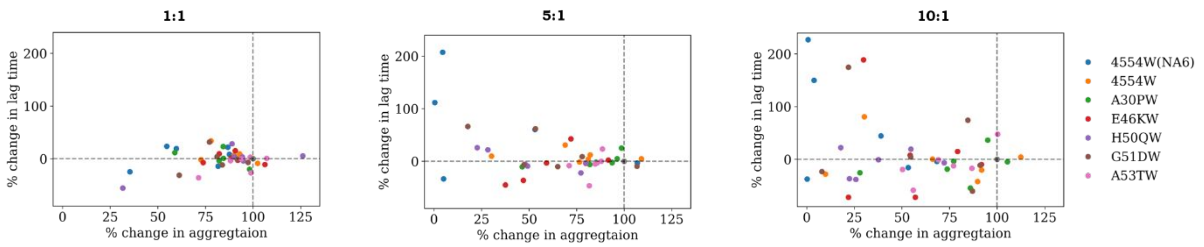

Figure S2*: *Scatter plot of aggregation vs. lag time for each peptide. Change in aggregation (ThT end point intensity) vs. change in lag time was plotted as a scatter plot to determine any correlation between these two parameters was observed. The coloured dots represent each peptide (4554W(N6A) (blue), 4554W (orange), A30PW (green), E46KW (red), H50QW (purple), G51DW (brown), and A53TW (pink)).

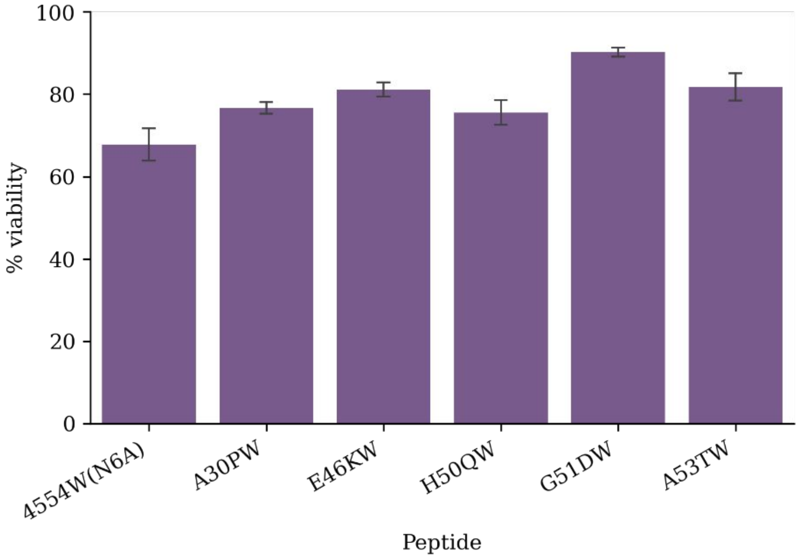

Figure S3: MTT of the peptide control samples. Each PCA-peptide (20 µM) was incubated on differentiated SH-SY5Y at 37 °C for 48 hr before cell toxicity was determine by MTT. Cell viability is shown as a percentage of the buffer control. Error bars represent the standard error.

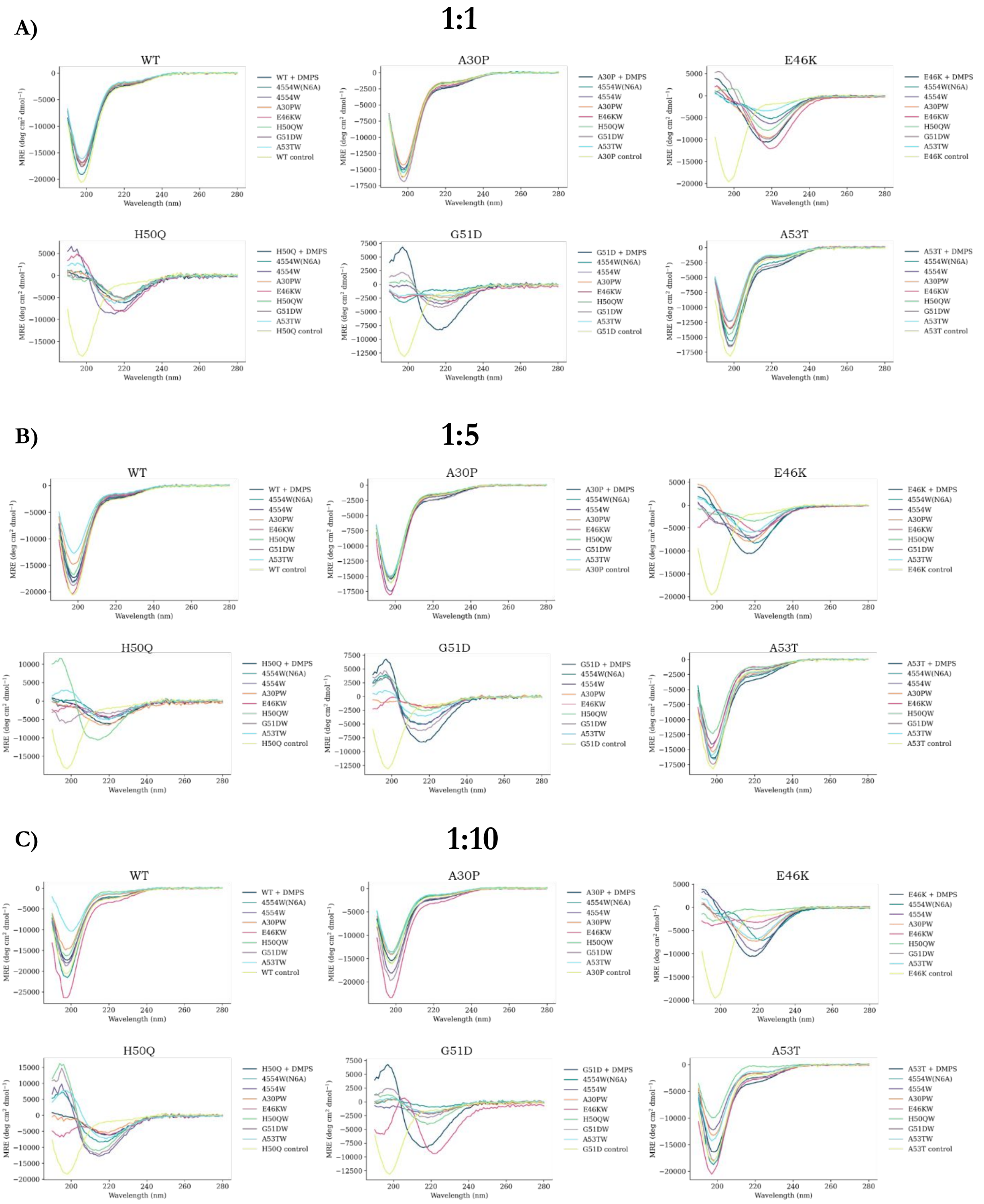

Figure S4: CD from the end point of the lipid-induced aggregation for each αS variant and each PCA-peptide. Aggregation assay end point samples were diluted 10-fold before the spectra was obtained. αS variant (with DMPS (dark blue), or without DMPS (yellow)) incubated with each PCA winner peptide (4554W(N6A) (teal), 4554W (purple), A30PW (orange), E46KW (pink), H50QW (green), G51DW (mauve), or A53TW (light blue). **A)** αS variant: peptide ratio is 1:1, **B)** αS variant: peptide ratio is 1:5, and **C)** αS variant: peptide ratio is 1:10. The spectra show the average of three repeats, are blanked against the assay buffer (20 mM sodium phosphate, pH 6.5), and the respective peptide control has been subtracted in order to view the changes to the αS structure.

αS purification overviews for each αS mutant

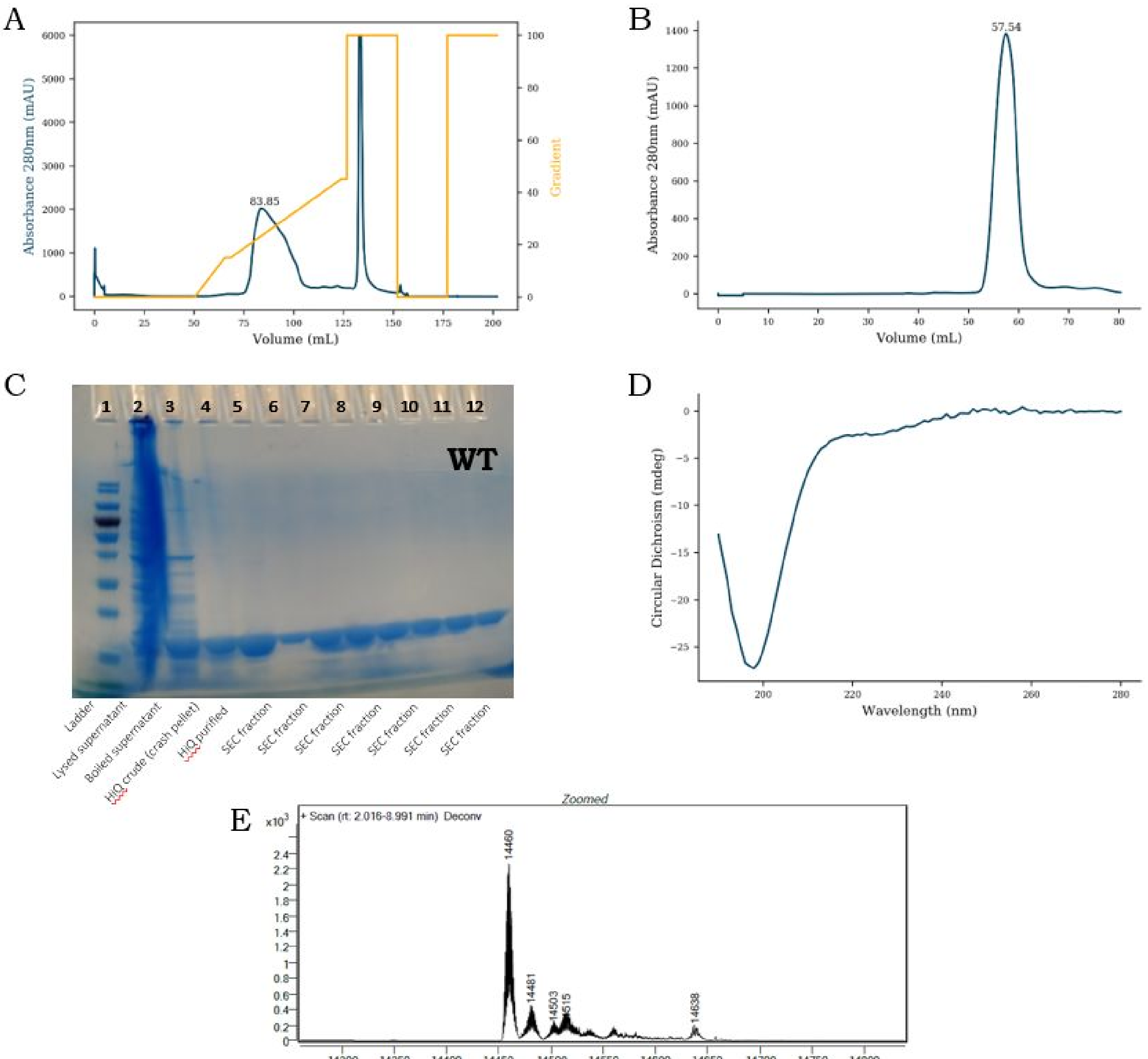

Figure S5: A) Anioinc exchange of αS(WT) showing the protein eluting at 83.85 mL. **B)** SEC chromatogram showing the monomer WT eluting at a peak centred around 57.54 mL. **C)** SDS-PAGE of the steps of the purification. **D)** CD spectra of the monomer protein confirming a random-coil secondary structure for the monomeric protein. **E)** MS of the purified protein. Expected mass: 14,460 Found: 14,460

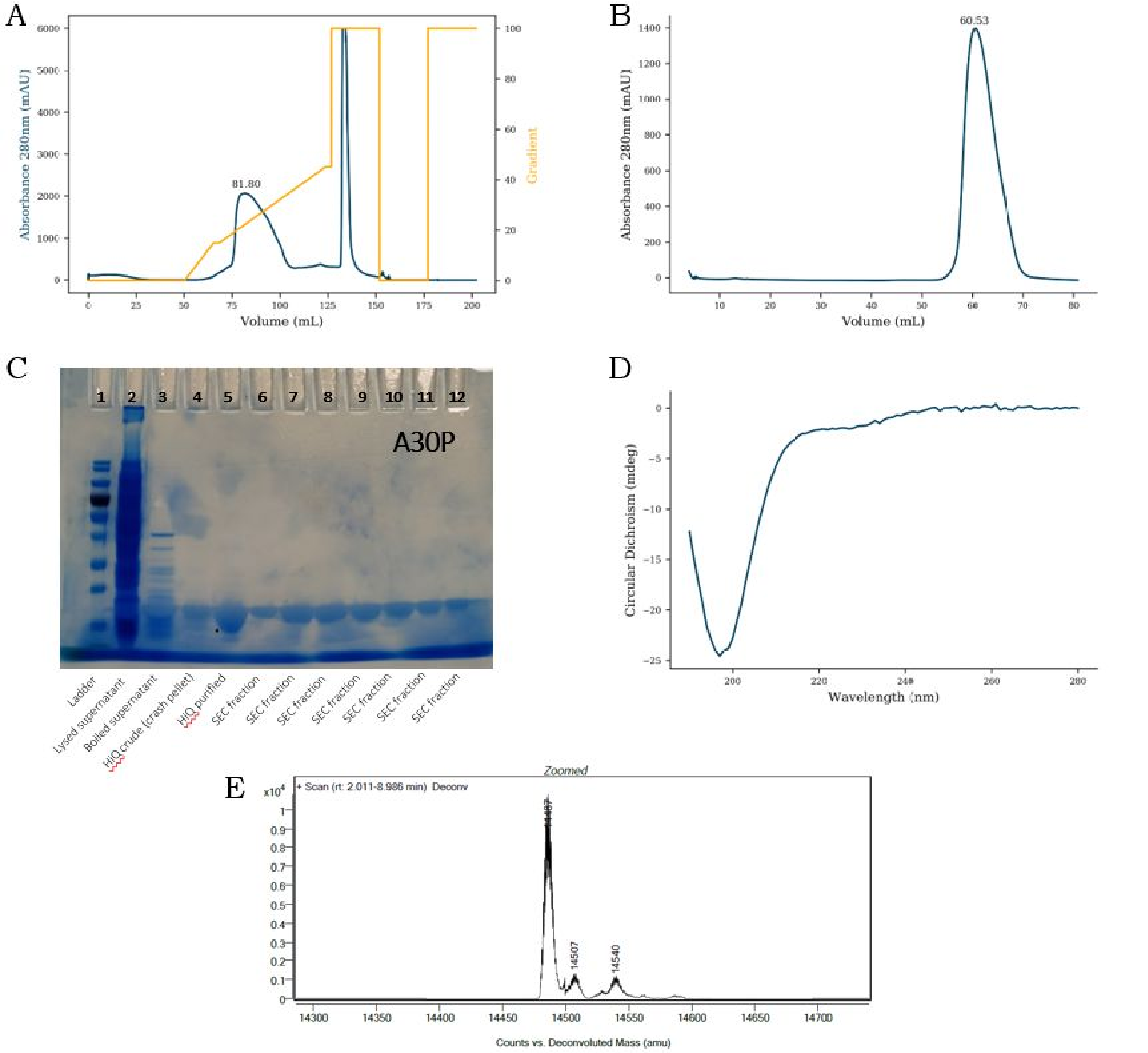

Figure S6: A) Anioinc exchange of αS(A30P) showing the protein eluting at 81.80 mL. **B)** SEC chromatogram showing the monomer A30P eluting at a peak centred around 60.53 mL. **C)** SDS-PAGE of the steps of the purification. **D)** CD spectra of the monomer protein confirming random-coil secondary structure for the monomeric protein. **E)** MS of the purified protein. Expected mass: 14,486 Found: 14,487

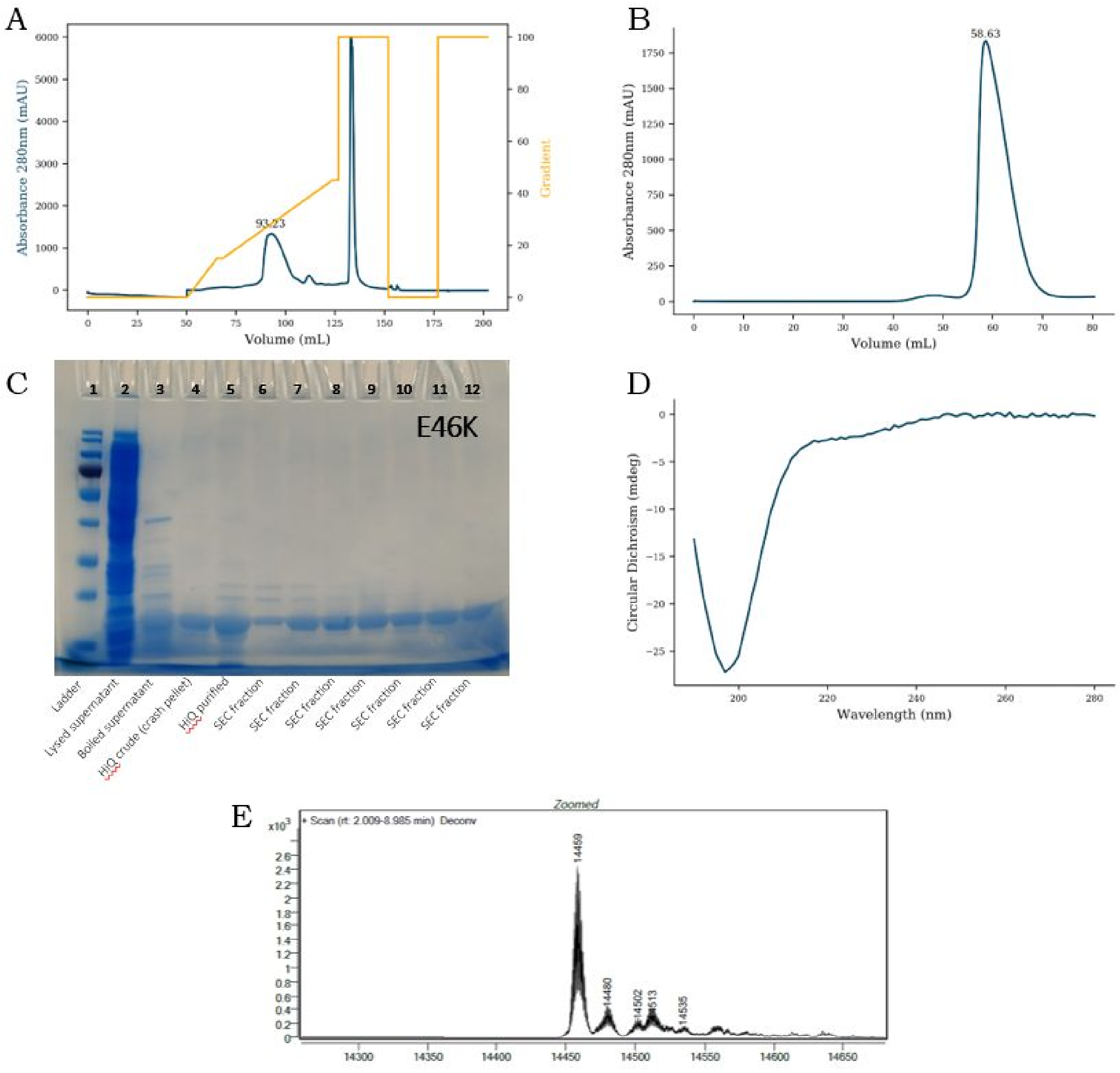

Figure S7: A) Anioinc exchange of αS(E46K) showing the protein eluting at 93.23 mL. **B)** SEC chromatogram showing the monomer E46K eluting at a peak centred around 58.63 mL. **C)** SDS-PAGE of the steps of the purification. **D)** CD spectra of the monomer protein confirming a random-coil secondary structure for the monomeric protein. **E)** MS of the purified protein. Expected mass: 14,459 Found: 14,459

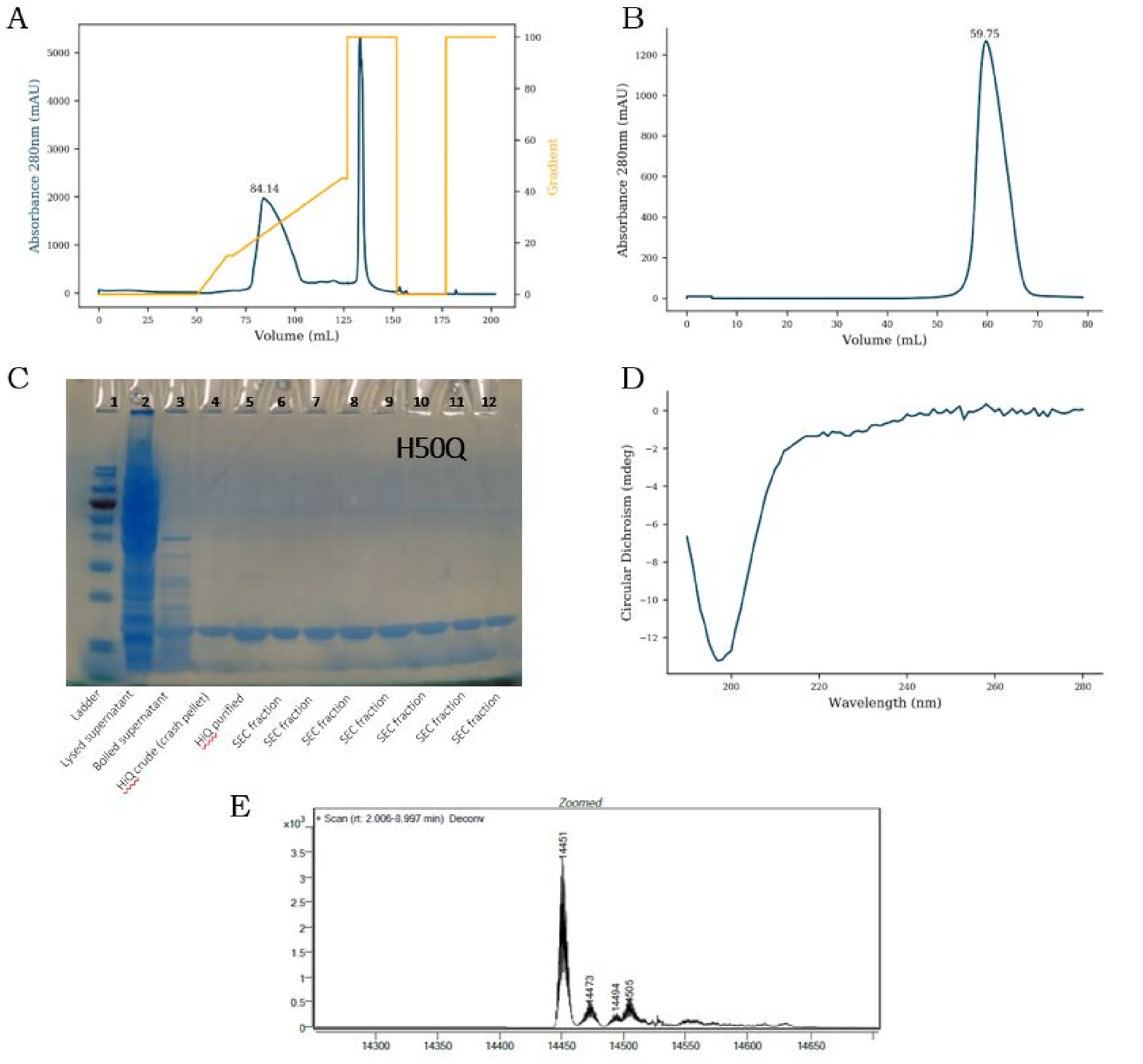

Figure S8: A) Anioinc exchange of αS(H50Q) showing the protein eluting at 84.14 mL. **B)** SEC chromatogram showing the monomer H50Q eluting at a peak centred around 59.75 mL. **C)** SDS-PAGE of the steps of the purification. **D)** CD spectra of the monomer protein confirming a random-coil secondary structure for the monomeric protein. **E)** MS of the purified protein. Expected mass: 14,451 Found: 14,451

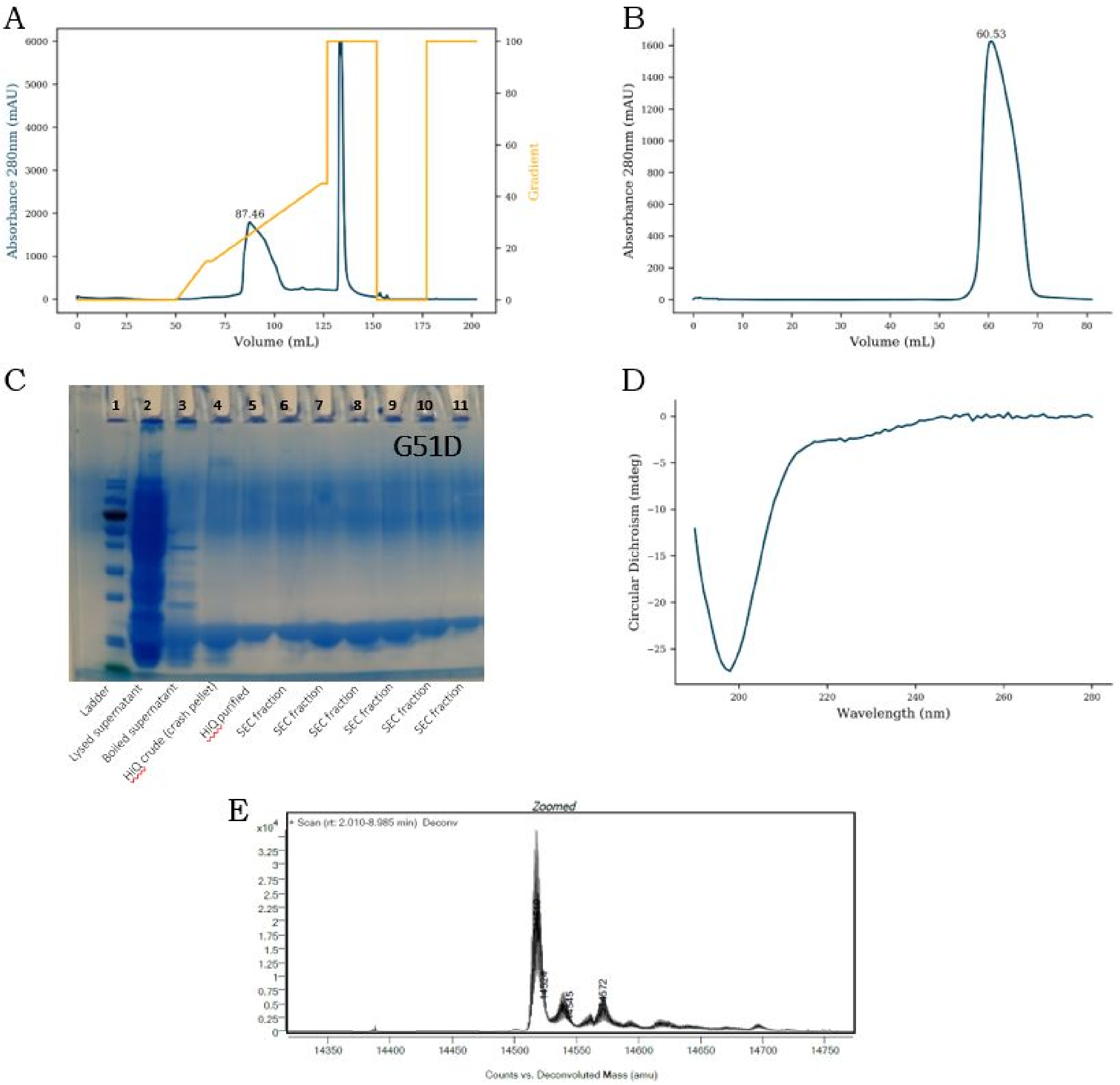

Figure S9: A) Anioinc exchange of αS(G51D) showing the protein eluting at 87.46 mL. **B)** SEC chromatogram showing the monomer G51D eluting at a peak centered around 60.53 mL. **C)** SDS-PAGE of the steps of the purification. **D)** CD spectra of the monomer protein confirming a random-coil secondary structure for the monomeric protein. **E)** MS of the purified protein. Expected mass: 14,518 Found: 14,519

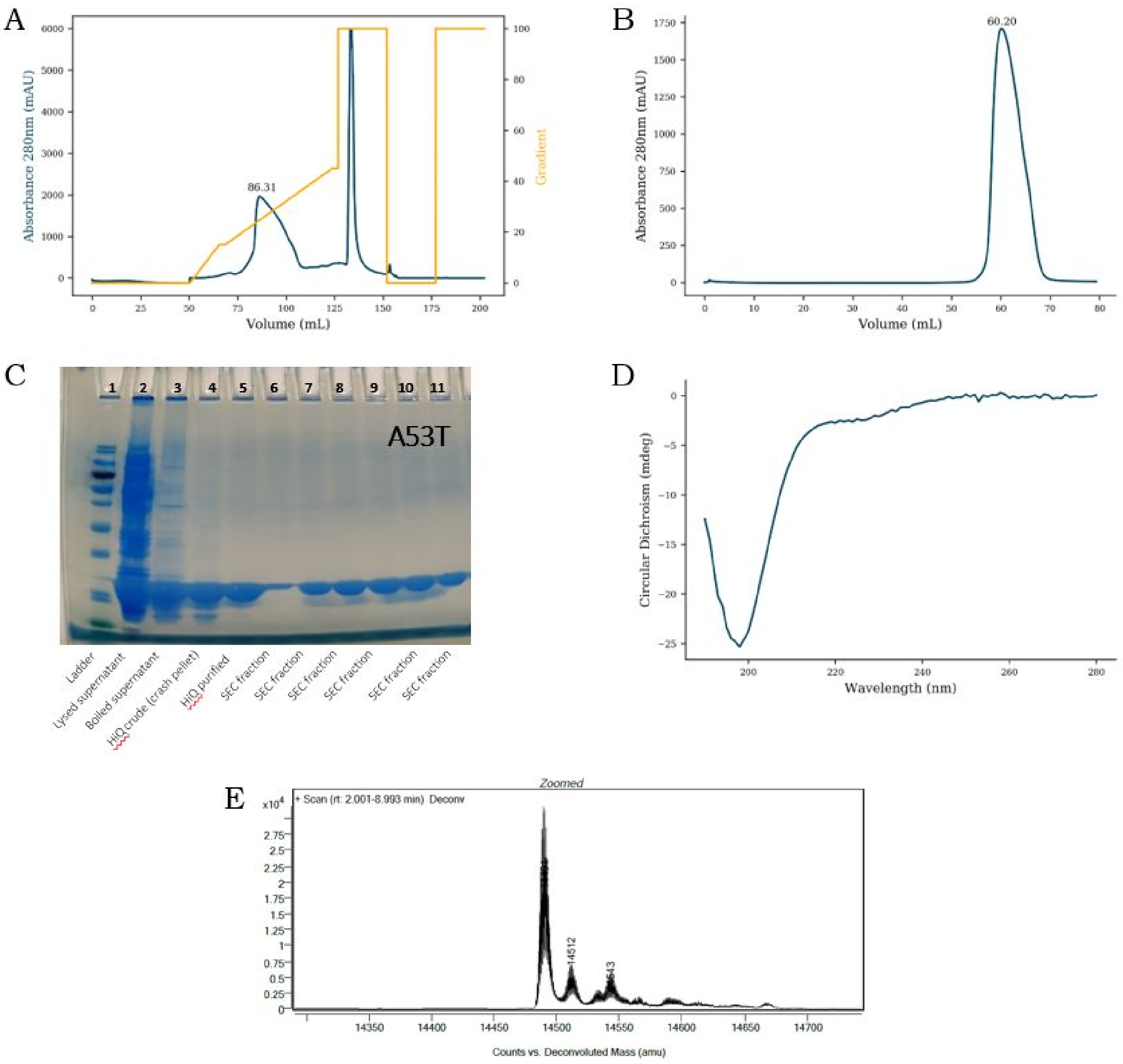

Figure S10: A) Anioinc exchange of αS(A53T) showing the protein eluting at 86.31 mL. **B)** SEC chromatogram showing the monomer A53T eluting at a peak centred around 60.20 mL. **C)** SDS-PAGE of the steps of the purification. **D)** CD spectra of the monomer protein confirming a random-coil secondary structure for the monomeric protein. **E)** MS of the purified protein. Expected mass: 14,490 Found: 14,491.
